# Progenitor cell‐derived basophils: A novel barcoded passive degranulation assay in allergic diseases

**DOI:** 10.1111/cea.14251

**Published:** 2022-11-16

**Authors:** Jiakai Wu, Rajia Bahri, Marina Tsoumani, Aida Semic‐Jusufagic, Clare S. Murray, Adnan Custovic, George V. Guibas, Miriam Bennett, Ran Wang, Gail Gauvreau, Ruth Cusack, Clare Mills, Silvia Bulfone‐Paus, Angela Simpson

**Affiliations:** ^1^ Division of Infection, Immunity and Respiratory Medicine, School of Biological Sciences University of Manchester Manchester UK; ^2^ NIHR Manchester Biomedical Research Centre, Manchester University NHS Foundation Trust Manchester Academic Health Science Centre Manchester UK; ^3^ Centre for Dermatological Research & Manchester Collaborative Centre for Inflammation Research (MCCIR), School of Biological Sciences University of Manchester Manchester UK; ^4^ National Heart and Lung Institute Imperial College London London UK; ^5^ Division of Respirology, Department of Medicine McMaster University Hamilton Ontario Canada

**Keywords:** allergy diagnosis, asthma, basophil, cat allergy, challenge tests, flow cytometry, peanut allergy

## Abstract

**Background:**

Effector cells assays provide an overall measure of responsiveness to allergen, but the lack of reliable and high‐throughput assays limits the clinical utility. We aimed to develop a high‐throughput basophil activation test based on human progenitor cell‐derived basophils (PCB) and investigate the role of PCB activation test (PCBAT) in allergic diseases.

**Methods:**

Progenitor cell‐derived basophils were differentiated from CD34^+^ progenitor cells and sensitized with sera from subjects sensitized to cat, peanut or atopic controls. Sensitized PCBs were stimulated with increasing concentrations of the corresponding allergens in vitro. Degranulation was assessed by measuring CD63 expression using flow cytometry. The correlations between PCBAT and clinical allergy were assessed.

**Results:**

Following passive sensitization of the mature PCBs with serum and allergen stimulation, an allergen specific dose‐dependent increase in CD63 expression was observed. Sera from subjects sensitized to cat (*n* = 35, of which 17 subjects had clinical reactivity quantified using inhaled allergen challenge), peanut allergic (*n* = 30, of which 15 subjects had clinical reactivity validated using double blind, placebo controlled food challenges [DBPCFC]), peanut‐sensitized but tolerant subjects (*n* = 5) were used to sensitize PCBs. PCBAT area under the curve (AUC) correlated with sIgE (*r* ^2^ = .49, *p* = .001) in subjects sensitized to cat (sIgE ≥ 0.35KU/L). The provocation concentration of inhaled cat allergen (PC_20_) correlated with PCBAT AUC (*r* ^2^ = .33, *p* = .016). In subjects sensitized to peanut, PCBAT AUC was highly correlated with sIgE to Ara h 2 (*r* ^2^ = .59, *p* < .0001). Peanut threshold cumulative dose during DBPCFC was negatively correlated with PCBAT AUC (*r* ^2^ = .57, *p* = .001) and IgE to Ara h1 (*r* ^2^ = .55, *p* = .007), but not with sIgE to whole peanut or Ara h2. All peanut‐sensitized but tolerant subjects showed no reaction to peanut on PCBAT.

**Conclusion:**

Progenitor cell‐derived basophils activation test is a high‐throughput assay, which correlates with clinical allergy and may confer a powerful alternative tool in allergy testing.

**Key Messages**

- Progenitor cell‐derived basophils are easy to set up, maturation takes <3 weeks.
- Progenitor cell‐derived basophil activation test allows batch test serum reactivity to allergen.
- Progenitor cell‐derived basophil activation test results are significantly associated with allergen challenge test results.

## 1 INTRODUCTION

Sensitization to inhalant allergens such as house dust mites, cats and dogs, is commonly associated with asthma, but is neither necessary nor sufficient for disease expression. Similarly, a positive skin prick test (SPT) or IgE test to a food does not equate to clinical food allergy, and false positive results are common. Challenge testing (either oral food challenge or inhaled allergen challenge) can be offered to patients, but is time consuming, carries the risk of severe reaction and is not suitable for all patients. Therefore, tests with superior diagnostic accuracy than IgE that are safe to use in all patients would be of value in clinical practice, especially amongst patients sensitized to many allergens.

Basophils and mast cells (MC) are the two primary effector cells in allergic responses,^1^ based on which several effector cell assays were developed.^4^, ^5^, ^12^, ^16^ Cellular degranulation triggers the release of preformed and newly synthesized mediators inducing a potent biological response in a sensitized person following allergen exposure.^2^ The two cell types may have different roles in an allergic response but this is poorly understood.^2^, ^3^ While basophils are found in the circulation, MC are localized in peripheral tissues. Hence, basophils are more accessible and are commonly used as cell models for studying allergy.^4^ However, basophils account for <1% of blood leukocytes, making purification a challenge. To obviate the need for purification, the basophil activation test (BAT) was developed using fresh whole blood, analysed immediately.^4^, ^5^ Following stimulation of whole blood with allergen (or control), the responsiveness of the basophils can be quantified using fluorochrome‐coupled antibody markers of basophil activation (e.g. CD63 and CD203c) by flow cytometry. The advantage of the BAT is that it takes account of many factors, which influence basophil responsiveness to an allergen such as IgG4/IgE ratio,^6^, ^7^ heterogeneity of sIgE to allergen components,^8^ medication^9^ and innate responsiveness of the cells.^10^ The disadvantages are that blood needs to be analysed immediately after being drawn,^5^ requiring the allergy clinic to have instant access to a staffed flow cytometry facility. In addition, 10%–20% of people carry ‘non‐releaser’ basophils, which are non‐responsive in the BAT, despite having clinical allergy.^11^ Consequently, this test is not generally available for clinical diagnostics, but used only in specialist laboratories for hymenoptera venom and drug allergy testing.

The passive BAT, which uses basophils from a donor that are passively sensitized with the serum from the patient, was developed as an alternative method that circumvents some of these problems.^12^ Stored serum samples from subjects can be analysed in batches, providing greater flexibility. This also allows humoral factors to be investigated separately from cellular factors.^7^ However, the donors' basophils must be stripped of endogenous IgE with a mild acid treatment before the cells can be passively sensitized with patient serum samples, which can damage the donor basophils and lead to auto‐basophil activation^13^ and reduced sensitivity.^14^ Due to these limitations, passive BAT has only been used in a few studies. Although the passively sensitized approach has also been used on basophilic cell lines such as RBL‐2H3, there are a number of disadvantages, including the gradual loss of cell responsiveness within weeks of cultures.^15^

To overcome the limitations of existing effector cell assays using basophils, this study we demonstrate a method of generating functional progenitor cell‐derived basophils (PCBs), and how they can be used to develop a reproducible, flow cytometry‐based basophil activation test (PCBAT) by passively sensitizing the PCBs with sera from cat or peanut‐sensitized patients. A detailed characterization of basophil differentiation, and demonstrate the functionality and reproducibility of this technique is provided. The potential clinical application of progenitor cell basophil activation test (PCBAT) is then explored by passively sensitizing the cells with sera from five groups of patients with allergic asthma and food allergy and testing degranulation to two allergens (cat and peanut).

## 2 METHODS

### 2.1 Study design

We developed a new high‐throughput BAT, using basophils generated from peripheral blood progenitor cells from healthy donors—the PCBAT. To assess the potential clinical utility, serum samples from cat or peanut‐sensitized patients were used to passively sensitize the basophils. The sensitized PCBs were incubated with increasing concentrations of cat or peanut allergens and basophil activation was assessed by measuring CD63 expression using flow cytometry. The associations between the degree of basophil activation and clinical characteristics of the study participants were then assessed.

### 2.2 Materials

#### 2.2.1 Development of progenitor cells‐derived basophil activation test (PCBAT)

##### Generating PCBs

Peripheral blood mononuclear cells (PBMCs) were isolated from leukocyte cones (NHS Blood and Transplant Centre, Manchester) using Ficoll density gradient centrifugation. CD34^+^ haematopoietic progenitor cells were isolated by a magnetic bead method according to manufacturer's instructions (MACS Miltenyl Biotec). Purified CD34^+^ haematopoietic progenitor cells were diluted to 1 × 10^5^ cells/ml and cultured in Stemspan™ supplemented with 10 ng/ml IL‐3, 100 ng/ml SCF, 50 ng/ml IL‐6, 5 mg/ml human LDL and penicillin/streptomycin (100 U/ml). This was day 0 of culture, cell density was then maintained between 2–5 × 10^5^/ml up to day 28 at 37°C with 5% CO_2_.

##### PCBs characterization

To monitor the differentiation process, the culture was sampled at day 7, 10, 16, 21 and 28. The cells were characterized using flow cytometry, immunofluorescence and metachromatic staining and by functional assay (PCBAT). This was repeated on two separate donors.

##### Flow cytometry

Cell staining was performed on a 96‐well plate using approximately 5 × 10^4^ cells/well. For PCB characterization, cells were stained with the following antibodies: CD63 (APC), CD123 (Percp‐Cy5.5), CD117 (BV605), CD203c (FITC), HLADR (eFluro450) and FcεRI (PE‐Cy7) for 20 min at 4°C. A detailed protocol including intracellular staining for 2D7 (PE) and fluorescence barcoding can be found in this article's online repository and gating strategy can be found in Figures S1 and S2.

##### Immunofluorescence staining

Cells were fixed in 4% paraformaldehyde and permeabilized with 0.1% tween and 10% goat serum. Cells were then stained in mouse anti‐BB1 antibody (1:10) followed by Alexa Fluor 555 goat anti‐mouse secondary antibody (1:200). Slides were mounted with fluoroshield mountant containing DAPI for cell nuclear staining and examined under a Leica DM IL LED microscope using Leica Application Suite software (Leica, UK).

### 2.3 Validation and performance of PCBAT

#### 2.3.1 Study subjects

Five groups with different clinical characteristics were identified and described in Table 1. All subjects provided written informed consent (North West ‐ Haydock Research Ethics Committee, REC reference 20/NW/0302; MAAS cohort registration: ICRCTN72673620 detailed elsewhere^16^; Hamilton Integrated Research Ethics Board, project number 5394).

**TABLE 1:** Demographic description of the study groups

			Sensitized subjects				Non‐sensitized control subjects			
Group number	Allergen used in PCBAT	Study group description	*n*	Age (years, Mean, ±SD)	Gender (% male)	sIgE (ku/L, Mean, ±SD)	*n*	Age (years, Mean, ±SD)	gender (% male)	sIgE (ku/L)
1	Cat	Sensitized to cat	18^a^	41.2 (15.2)	72.2%	12.10 (24.52)	6^a^	61.0 (21.3)	33.3%	<0.4
2	Cat	Asthma, on Omalizumab, sensitized to inhalant allergens	4^a^	45.9 (14.7)	100%	36.05 (47.52)	N/A			
3	Cat	Clinical reactivity quantified with inhaled allergen challenge to cat	17^b^	38.1 (15.6)	52.9%	N/A	6^a^	49.5 (9.9)	33.3%	<0.4
4	Peanut	Doctor diagnosed peanut allergy (clinical reactivity quantified using oral food challenge for 15 subjects, see OLS for details)	30^a^	24.5 (7.3)	46.7%	36.99 (38.60)	4^a^	44.8 (5.1)	50%	<0.4
5	Peanut	Sensitization to 1 or more peanut allergen components, but self‐reported ingestion indicating oral tolerance	5^c^	16.0 (1.2)	40%	N/A	N/A			

^a^ Patients recruited from ManARTS biobank (Rec reference: 20/NW/0302).

^b^ From McMaster University, Ontario, Canada.

^c^ From the population birth cohort—Manchester Asthma and Allergy Study (MAAS, registration: ICRCTN72673620) detailed elsewhere.^16^

#### 2.3.2 Measurement of sensitization to cat and peanut allergens

Serum sIgE to cat was measured in Groups 1 and 2 subjects and sensitization was defined as sIgE ≥0.35 kU/L. SPT to cat allergen were performed in Group 3 subjects with a positive test defined as a wheal of 3 × 3 mm or greater. sIgE to peanut and peanut components allergens Ara h 1, 2, 3, 8, 9 (Immunocap, ThermoFisher Scientifc, Sweden) were measured in Group 4 and sIgE to Ara h 1, 2, 3, 6, 8, 9 were measured using ISAC (ThermoFisher Scientifc, Sweden) in Group 5 subjects (a positive result was defined as sIgE > 0.3 ISU‐E).

#### 2.3.3 Inhaled cat allergen challenge

Seventeen cat sensitized adults underwent inhaled cat allergen challenge at McMaster University, Ontario, using Cockcroft methods described previously^17^ (see Online Repository). Briefly, participants inhaled cat allergen at increasing concentrations until the forced expiratory volume within 1 s (FEV_1_) dropped by ≥20% from baseline. The provocation concentrations eliciting 20% fall in FEV1 were calculated (PC_20_).

#### 2.3.4 Oral peanut challenge

Of 30 physician‐confirmed peanut allergic patients, 15 underwent double blind, placebo controlled food challenges (DBPCFC) to peanut^18^ (challenge protocol in the article's Online Repository). Participants ingested increasing quantities of peanut protein until objective signs of an allergic reaction were shown. The cumulative dose of peanut required to show first objective sign was used as a measure of clinical reactivity to peanut allergen.

#### 2.3.5 PCBAT

Progenitor cell‐derived basophils were sensitized with either 20% patients' sera or with human myeloma IgE (1 μg/ml) overnight at 37°C with 5% CO_2_. On the follow day, allergens extracts of different dilutions were freshly prepared from the stock using fully supplemented culture media. Sensitized PCBs were activated by incubating with serial dilutions of extracts (roasted peanut extracts or cat allergen) in the presence of serum for 30 min at 37°C. Anti‐IgE (1 μg/ml) and ‘medium only’ was included for every subject as positive and negative controls, respectively. PCBs were identified by staining the cells with CD203c^+^(FITC) and FcεRI^+^(PE‐Cy7). CD63 (PE) was used as a degranulation marker. After cells were stained with antibodies and viability dyes, Fluorescent barcoding (16‐plex) was performed using methods previously described,^19^ detailed barcoding protocol can be found in this article's online repository. A minimum 5% of CD63 positive cells were required to indicate a positive PCBAT response. To depict the responsiveness of the PCBAT, we present results as AUC for CD63 expression at increasing allergen concentrations.

#### 2.3.6 Statistics

Demographic variables were presented as means and standard deviation. The AUC was calculated using the trapezoidal rule on logarithmically transformed allergen concentrations to quantify the responsiveness of a degranulation assay, as previously described.^20^ Methods for EC50, CDsens and CDmax calculation are described in this articles' Online Repository. Correlations were assessed using Spearman R test for non‐parametric data and Pearson R test for normally distributed data (SPSS v22, IBM, Armonk, USA). A 2‐sided *p* value ≤ .05 was considered statistically significant.

## 3 RESULTS

### 3.1 PCBAT development

#### 3.1.1 Characterization of PCBs maturity

**FIGURE 1:**
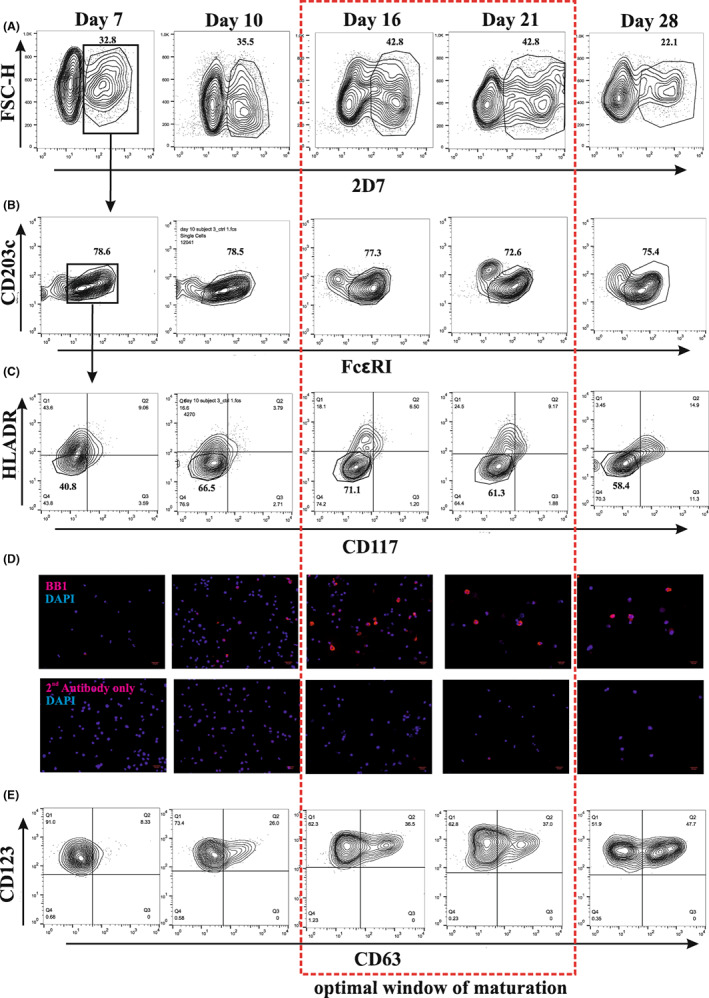
Characterization of PCBs differentiation during day 7–28 of culture. (A–C) Representative figures for non‐stimulated controls showing expression of basophil selection markers 2D7^+^/FcεRI^+^/CD117^−^/HLADR^−^ during culture. (D) Parallel analysis of BB1 expression by immunofluorescence staining. (E) Degranulation capability by stimulating IgE sensitized cells with anti‐IgE during culture. Gating strategies were the same as illustrated in A–C.

Human primary PCBs were differentiated from CD34^+^ haematopoietic progenitors following 28 days of culture. The maturity of PCBs was demonstrated using four methods ‐ immunophenotyping (Figure 1A–C), immunofluorescence staining (Figure 1D), functional tests (Figure 1 E) and morphological study (Figure S3) for PCBs obtained from two donors (donor A in Figure 1 and donor B in Figure S4).

Mature PCBs were defined as 2D7^+^/FcεRI^+^/CD117^−^/HLADR^−^ cells. The proportion of cells expressing the basophil marker 2D7 increased from day 7 (32.8%) peaked at day 16 (42.8%) and fell to 22.1% by day 28 (Figure 1A). When the 2D7^+^ cells were gated for FcεRI^+^, the majority (~80%) were positive through the culture (Figure 1B). Furthermore, as the cells matured, the highest negativity for CD117^−^ and HLADR^−^ were seen at day 16 (Figure 1C). However, by day 28, the cells started to gain CD117 receptor expression, an additional indication that the culture was losing basophilic characteristics.

We performed immunofluorescence staining with another basophil marker, BB1. The BB1 positive cells were faintly visible at day 7, but clearly visible by day 16 and remained visible to day 28 (Figure 1D).

We tested the PCBs' ability to degranulate upon engagement of the FcεRI by sensitizing them with human myeloma IgE followed by anti‐IgE stimulation. The percentage of basophil activity, as measured by CD63 expression, increased as the cells matured, such that by day 16, 36.5% of cells showed degranulation (Figure 1E). This was repeated with PCBs from 4 other donors and showed a consistent percentage of activation between donors at day 16 (mean ± SEM: 41.31% ± 3.34, Figures S5A,B). The maturation of PCBs and degranulation capability at each stage was very similar between the two donors (Figure 1, Figures S4 and S5C). Unstimulated control at each stage can be found at Figure S5E. Representative figures for cell viability through culture were shown at Figure S5D.

Using this method, an average yield (from nine donors) of 2.27 × 10^7^ cells (9 × 10^6^ ‐ 4.8 × 10^7^, minimum‐maximum) could be achieved at day 16 of culture, of which 25%–50% were basophils (Figure S8). May‐Grünwald Giemsa staining performed on day 16 cells showed heavily granulated cells (Figure S3).

The combined use of immunophenotyping, immunofluorescence, morphological characterization and functional tests suggested that the optimal window for PCBs was between day 16 and day 21. After day 21, cells began to lose basophilic features but still retained a high response to anti‐IgE following IgE sensitization (Figure 1A,E).

#### 3.1.2 High‐throughput PCBAT with fluorescent barcoding

To increase the throughput, we incorporated fluorescent barcoding to the PCBAT (Figures S6 and S7). We simplified the gating strategies for selecting PCB population to minimize the interference between fluorescence dyes and the antibody panel. We selected CD203c^+/^FcεRI^+^ cells for the degranulation assay, as CD203c^+^ cells were >99% 2D7^+^ (Figure S8). In addition, amongst CD203c^+^ cells, only FcεRI^+^ population could degranulate in response to FcεRI crosslinking (Figure S9).

#### 3.1.3 PCBAT in cat allergy

Patient demographics and clinical characteristics are summarized in Table 1. PCBAT, sIgE and SPT results are presented in Tables S1–S4. All samples responded to positive control stimulant (anti‐IgE) and not to negative control stimulant (medium only). Results for sensitivity (EC50 and CDsens) and reactivity (CDmax) of the PCBAT are presented in Tables S1–S5.

##### PCBAT and sIgE in cat sensitized asthma patients

**FIGURE 2:**
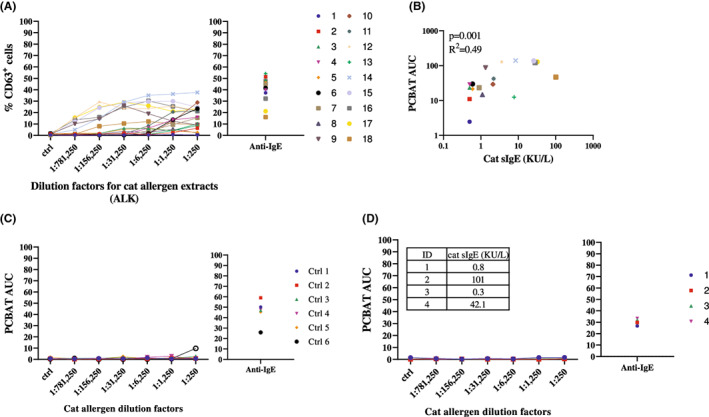
PCBAT with cat allergen extracts on cat sensitized but clinical reactivity not validated subjects. PCBAT using cat allergen extracts from ALK (dilution factor 1:250; 1:1250; 1:6250; 1:31,250; 1:156,250 and 1:781,250) were performed on (A) 18 cat sensitized but clinical reactivity not validated subjects; (B) the relationship between the PCBAT AUC from the sensitized groups and the corresponding sIgE level were shown in scatter plot. (C) PCBAT using cat allergen extracts were also performed on six atopic but non‐cat sensitized subjects and (D) 4 subjects who are sensitized to cat allergen but under omalizumab treatment, spearman test and *R* square was calculated and *p* < .05 were considered significant.

All but one (94.4%) cat sensitized patients (Group 1, *n* = 18) showed positive responses in PCBAT (Figure 2A). There was a significant correlation between PCBAT AUC and sIgE to cat (*r* ^2^ = .49, *p* = .001; Figure 2B). Five of the six control subjects with asthma who were not sensitized to cat showed a negative response in PCBAT (Figure 2C). One control subject showed a weak positive response at the highest concentration. No response to cat allergen was observed in PCBAT in patients who were receiving omalizumab (*n* = 4, of which three were sensitized to cat [Group 2, Figure 2D]).

##### PCBAT in predicting inhaled cat allergen challenge

**FIGURE 3:**
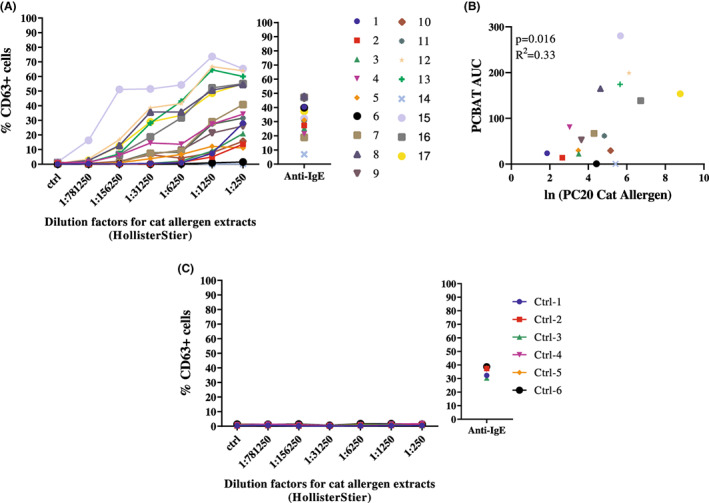
PCBAT with cat allergen extracts on cat allergic subjects validated with inhalant allergen challenge. PCBAT using cat allergen extracts from HollisterStier (dilution factor 1:250; 1:1250; 1:6250; 1:31,250; 1:156,250 and 1:781,250) were performed on (A) 17 clinal reactivity validated cat allergic subjects; (B) Scatter plot showed the relationship between the PCBAT AUC from the allergic groups and the corresponding natural log transformed dosage response slope. (C) PCBAT using cat allergen extracts were also performed on six atopic but non‐cat sensitized subjects, spearman test and *R* square was calculated and *p* < .05 were considered significant.

Seventeen adults demonstrated airway reactivity during inhaled cat allergen challenge (Group 3). The median (IQR) of SPT wheal size was 6 (4–7.6) mm (Table S4) and the range for allergen PC_20_ was large (median [IQR]: 102.5 [32.8–286.9] BAU/ml; Figure S10B). Of the 17 subjects, 15 showed a positive response on PCBAT (Figure 3A), two subjects showed no response. PCBAT AUC significantly correlated with ln(PC_20_ cat allergen) (*r* ^2^ = .33, *p* = .016; Figure 3B) but SPT wheal sizes did not (*p* = .230). All control subjects (*n* = 6) showed no response in PCBAT (Figure 3C).

#### 3.1.4 PCBAT in peanut allergy

##### PCBAT in confirmed peanut allergy

**FIGURE 4:**
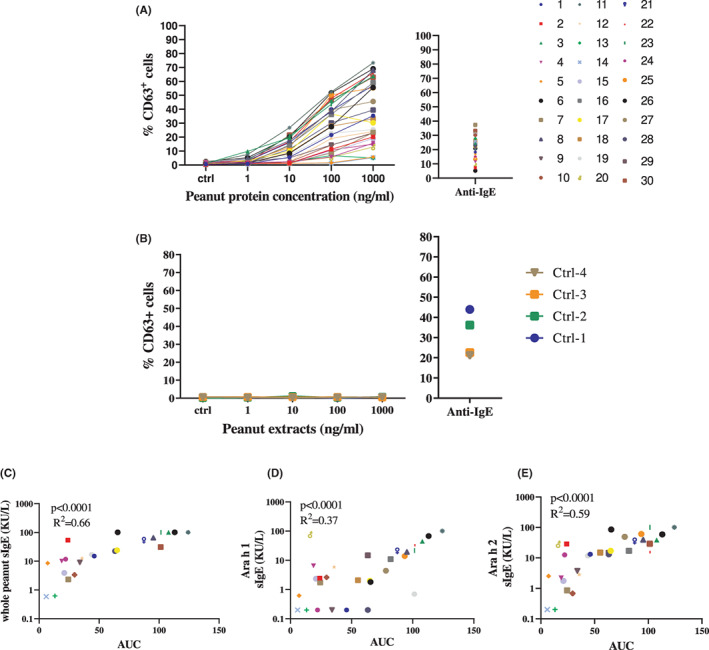
PCBAT with peanut allergen extracts on physician diagnosed peanut allergic patients. PCBAT using peanut allergen extracts (1, 10, 100 or 1000 ng/ml) were performed on (A) 30 subjects with physician diagnosed peanut allergy, and (B) on four atopic but non‐peanut‐sensitized subjects. The relationship between the PCBAT AUC from the sensitized groups and the corresponding whole peanut sIgE level Ara h 1 sIgE level and Ara h 2 sIgE level were shown in scatter plot (C–E, respectively). Spearman test and *R* square was calculated and *p* < .05 were considered significant.

All adults with physician diagnosed peanut allergy (*n* = 30, Group 4) showed positive responses in PCBAT, which were dose‐dependent (Figure 4A), and all negative control subjects (*n* = 4) did not respond to peanut on PCBAT (Figure 4B). There was a significant correlation between PCBAT AUC and sIgE to whole peanut and Ara h 1, 2 (Figure 4C–E), 3 and 6 (Figures S11A,D) but not with sIgE to Ara h 8 and 9 (*p* > .7; Figures S11B,C).

**FIGURE 5:**
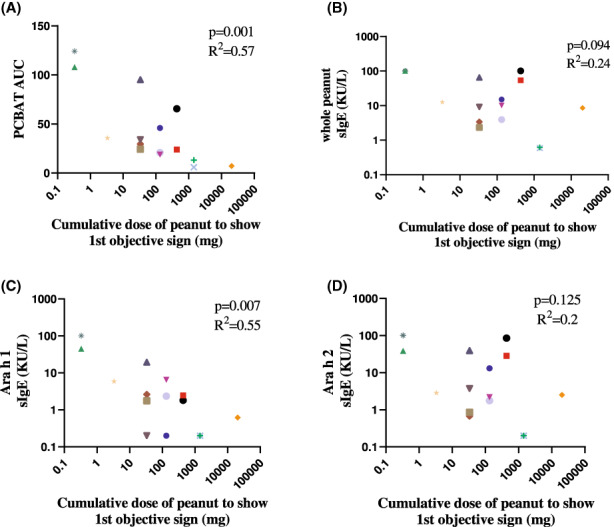
PCBAT with peanut allergen extracts on peanut allergic subjects validated with oral peanut challenge. Of the 30 peanut allergic subjects performed with PCBAT, 15 underwent oral food challenge. Scatter plot showed the relationship between cumulative peanut dose to show first objective sign and PCBAT AUC (A) or sIgE to whole peanut (B), Ara h 1 (C) and Ara h 2 (D). Spearman test and R square was calculated and *p* < .05 were considered significant.

Of the 30 subjects with physician diagnosed peanut allergy (Group 4), half had confirmed peanut allergy following DBPCFC. Subjects who showed a higher PCBAT AUC reacted at a lower dose of peanut during DBPCFC (*r* ^2^ = .57, *p* = .001, Figure 5A). A significant negative correlation was also observed between sIgE to Ara h 1 and oral food challenge results (*r* ^2^ = .55, *p* = .007), but not with IgE to whole peanut (*p* = .094) or Ara h 2 sIgE (*p* = .125) (Figure 5B–D).

##### PCBAT in peanut‐sensitized but tolerant subjects

**FIGURE 6:**
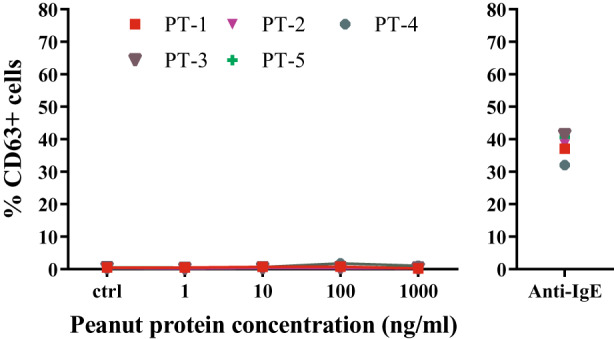
PCBAT with peanut allergen extracts on peanut‐sensitized but tolerant subjects PCBAT using peanut allergen extracts (1, 10, 100 or 1000 ng/ml) were performed on 5 peanut‐sensitized but tolerant subjects. Anti‐IgE was used as positive control stimulant for all tested serum samples. Spearman test and *R* square was calculated, *p* < .05 were considered significant.

Within a population‐based birth cohort, we identified five subjects (Group 5) who were sensitized to 1 or more peanut allergen component (Ara h 1, 2, 3 or 6; Table S6), but self‐reported tolerance to peanut. No subjects showed responsiveness to peanut extracts in PCBAT even at the highest concentration (Figure 6).

## 4 DISCUSSION

We have developed a high‐throughput progenitor cell‐derived basophil activation test (PCBAT), which was a better predictor of clinical reactivity to cat and peanut allergen (as measured on challenge testing) than conventional markers of allergy such as SPT or allergen specific IgE. PCBAT can be used with stored serum, removing the need for immediate access to expensive flow cytometry facilities not generally available in the clinic. By passively sensitizing basophils with sera from our well characterized patient populations then culturing with the relevant allergen, we demonstrated dose‐dependent and allergen specific basophil activation with wide variability in trajectories. PCBAT allows humoral factors to be investigated separately from cellular factors.

### 4.1 Technical aspects of the PCBAT

The combined use of flow cytometry and immunostaining suggest the optimal window for maturation for this culture protocol was between day 16 and 21, consistent with the previous studies.^22^ Our culture was highly enriched for mature basophils (25%–50%), enabling high‐throughput barcoding and improved efficiency. Due to the lack of standardized cat allergen extract, we used cat skin prick test solution for PCBAT (ALK for Groups 1 and 2), and applied dilution factors for all the relevant abscissa. Where patients had undergone inhaled cat allergen challenge (Group 3), the same cat allergen extract for PCBAT was used (HollisterStier). Since the concentration of Fel d 1 in ALK allergen solution was twice that of HollisterStier (Figure S12), PCBAT comparison was not made between groups.

### 4.2 PCB responsiveness and sIgE levels

The PCBAT reaction was allergen specific, showing a dose‐dependent response with good association with levels of corresponding sIgE. In addition, patients receiving omalizumab treatment showed completely muted responsiveness in the PCBAT, in accordance with a previous study.^22^ However, one subject, with low but just‐detectable sIgE to cat (0.5 ku/L) did not show responsiveness in PCBAT; this patient was not on omalizumab treatment. As this subject had not undergone allergen challenge, it remains unclear whether this subject showed airway reactivity to cat allergen. One subject with sIgE to cat of <0.35 ku/L, who had a positive dog sIgE (5 ku/L), showed minor degranulation on PCBAT but only at the highest concentration. We speculate that this weak response to cat allergen might reflect cross‐reactivity between cat allergen and dog sIgE, which has been previously reported.^23^, ^24^, ^25^ We have also found quantifiable traces of Can f 1 in both cat allergen extracts used (Figure S12).

### 4.3 Validation of PCBAT in clinical allergy

We validated PCBAT using two cohorts of patients who underwent inhaled cat allergen challenge or double blind placebo controlled oral food challenge of peanut.

Although a significant association was observed between PCBAT AUC and allergen PC_20_, two subjects showed negative results in the PCBAT but reacted to inhaled cat allergen. It is noteworthy that as the blood samples were collected within 2 years of the inhaled allergen challenges, during which changes in allergen sensitivity and exposure is possible. Further prospective studies using sera collected near the time of inhaled allergen challenge and also sera from people with moderate and severe asthma is mandatory to confirm its role in predicting clinical reactivity.

We explored PCBAT in peanut allergy as an exemplar of an allergic disease where clinical reactivity is not reliably predicted by serum sIgE, and oral food challenges are necessary to confirm allergy and quantify thresholds of responsiveness. All 30 patients with physician diagnosed peanut allergy showed a positive response in PCBAT; in addition, PCBAT showed a better correlation with threshold cumulative dose ingested during DBPCFC when compared with serum sIgE to whole peanut extract or to Ara h 2 and 3. Also, PCBAT were negative in a small number of patients that reported regularly eating peanuts but had positive sIgE to 1 or more peanut allergen component (Ara h 1, 2, 3 or 6). This suggests PCBAT may be a useful tool in differentiating individuals with peanut allergy from those who are sensitized but tolerant, but further testing on more subjects would be required for the evaluation of diagnostic and predictive powers. Also, it will be important to study the role of PCBAT in subjects sensitized only to Ara h 8 or Ara h 9, but stimulation would need to be with an extract containing high concentrations of these allergens, which occur at low concentration in the native food.

### 4.4 Limitations

One of the major limitations of PCBAT is that the assay only focuses on the humoral factors whereas in a BAT where patient's own blood was used, the response is a combined effect from humoral and cellular factors.^26^, ^27^, ^28^ Therefore, PCBAT might not accurately simulate an allergic response as it does not take account of the between individual differences in native basophil reactivity. Although PCBAT has been evaluated against conventional diagnostic tests in allergy and demonstrates clear technical advantages over other basophil‐based effector cell assays, further studies are needed to directly compare the diagnostic powers of PCBAT with these assays and establish its clinical usefulness. Finally, we validated PCBAT in cat and peanut‐sensitized individuals, but its role in other allergies remains to be elucidated.

## 5 CONCLUSION

By generating progenitor cell‐derived basophils in high‐yield we have developed a flow cytometry‐based basophil activation test for use with stored serum, which can be used to assess reactivity to both food and inhalant allergens. By incorporating fluorescent barcoding, we have increased the throughput of the assay significantly. We identified wide variability in trajectories of response to allergen in different subjects, and responses were muted in the presence of the anti‐IgE treatment omalizumab, indicating that this test better reflects the overall immune milieu rather than just specific IgE. For subjects who had undergone oral food challenge to peanut, we were able to use results of PCBAT to predict clinical reactivity to peanut. The correlation of PCBAT to clinical reactivity to inhaled cat allergen may represent a safe and robust way of identifying those asthmatics who might benefit from interventions for ongoing cat allergen exposure. Although further evaluation is required, this proof of concept study indicates that this test may have a role in food and inhalant allergy testing as a means of identifying clinically important sensitizations.

## AUTHOR CONTRIBUTION

JW, AS and SBP designed the study and wrote the manuscript. JW, RB and MT designed experimental protocols. JW performed the experiments and analysis. ASJ and GVG performed OFC and subject selection of peanut tolerant and peanut allergic groups. GG and RC performed cat allergen inhalant challenge and MB and RW conducted subsequent data analysis and discussion. CM is the PI for the iFAAM study and provided peanut allergen. CSM and AC are the PIs of MAAS cohort and contributed to the editing of the manuscript. All authors contributed in revising the manuscript.

## CONFLICT OF INTEREST

The authors have no conflicts of interest to declare that are relevant to the content of this article.

## Supporting information


Appendix S1.

## Data Availability

All data generated or analysed during this study are included in this published article (and its supplementary information files).
