# Supplementary material for: Progenitor cell‐derived basophils: A novel barcoded passive degranulation assay in allergic diseases
Source: Clin Exp Allergy. 2022 Nov 16;53(4):405–16. doi: 10.1111/cea.14251 (PMC10946533; doi:10.1111/cea.14251)
Supplement: Supplementary file 1 — Appendix S1. [file CEA-53-405-s001.docx]

## Online Repository

## Materials

## Allergen extracts used: Peanut extract at 1mg/ml concentration was provided by Professor Clare Mills. Cat allergen for testing patient group 1 and 2 were skin test solutions obtained from ALK (ALK555, Reading, UK). Cat allergen for testing group 3 were the same cat allergen used in the inhaled cat allergen challenge (Standardized Cat Hair, manufactured by Jubilant HollisterStier (Spokane, WA, USA).

## Reagents, antibodies, cytokines and kits used: Anti-basogranulin (BB1) mouse monoclonal antibody was a gift from Dr Andrew Walls. May-Grünwald solution and DPX (Sigma Aldrich); CD34 MicroBead kit (MACS Miltenyl Biotec); LIVE/DEAD® Fixable Blue Dead Cell Stain Kit (Life technologies); Intracellular Fixation & Permeabilization buffer set (eBioscience); human myeloma IgE (Merck Millipore); goat anti-IgE (Insight Biotechnology Ltd); Fluoroshield mount with DAPI (Abcam); stem cell factor (SCF), IL-3 and IL-6 (PeproTech); Stemspan^TM^ and human LDL (STEMCELL technologies); CD63-APC (H5C6); CD63-PE (H5C6); CD117-BV605 (104D2); HLADR-eFluro450 (LN3); FcεRI-PE-Cy7 (AER-37 (CRA-1)); and 2D7-PE (Biolegend); Alexa Fluor 555 goat anti-mouse secondary antibody (Abcam); Pacific Blue Succinimidyl Ester (Thermo Fisher), Alexa Fluor 700 NHS Ester (Thermo Fisher). Can f 1 ELISA kit (INDOOR biotechnologies, EPC-CF1)

# Methods

## Peanut oral food challenge (OFC)

Peanut allergic subjects with a positive skin prick test result to peanut (wheal ≥ 3 mm) and a physician diagnosis of peanut allergy were prospectively recruited from allergy clinics (Manchester University NHS Foundation Trust) and research databases to have an oral food challenge to peanut. Subjects gave written informed consent to undergo a double blind-placebo-controlled oral food challenge (DBPCOFC) with peanut. The challenges (active and placebo) were conducted on 2 separate days at least one week apart. Subjects were randomized to receive either peanut or placebo containing cookies on the first day and then received the other challenge meal on the second day. The challenge involves the subject ingesting progressively larger quantities of peanut protein (or placebo) at 30 minute intervals throughout the day, whilst being monitored continuously for the development of symptoms and signs of a possible allergic reaction. The dosing schedule used was that developed for the Europrevall study dose schedule (3, 30 and 300 μg; 3, 30, 100, 300 mg; 1 and 3 g peanut protein) (1).

Challenges results were typically considered positive, and dosing was stopped after the dose that provoked the first objective sign. Objective signs leading to discontinuation of the challenge were blisters of oral mucosa, erythema, urticaria, angioedema, rhinitis, conjunctivitis, wheeze, cough, hoarseness, stridor, laryngeal oedema, diarrhea, emesis, tachycardia, blood pressure drop of at least ≥20%, significant change in mental state, or severe persistent subjective symptoms lasting more than 45 minutes such as severe itching of palms, soles, head or severe gastric/abdominal pain. If all DBPCOFC doses were negative, patients underwent open challenges with 16g of salted peanuts. In case of allergic reaction subjects were treated and the challenge was stopped.

## Inhaled cat allergen challenge

Seventeen cat sensitized adults underwent inhaled cat allergen challenge at McMaster University, Ontario. The Cockcroft equation (2) was used to calculate the predicted provocation concentration (PC_20_) of inhaled allergen to cause a 20% drop from baseline lung function (as measured by the forced expiratory volume in 1 second, FEV_1_). This calculation is based on reactivity to methacholine (all had airway reactivity to methacholine with PD_20_ ≤16mg/ml), and skin prick test titration to cat allergen (which identifies the weakest dilution of cat allergen extract which generates a 2x2 mm wheal on skin prick test). A safe starting dose of inhaled cat allergen was chosen at three doubling doses below that predicted allergen PC_20_.

During the allergen inhalation challenge, sequential doubling concentrations of cat allergen were inhaled with 2 minutes of tidal breathing with an English Wright nebuliser (Roxon, Quebec, Canada). FEV_1_ was measured 10 minutes after each dose. Administration of allergen stopped once FEV_1_ dropped 20% from baseline. Dose response slope (DRS) was calculated using the natural log of the maximum percentage drop in FEV_1_ during 0-2 hours after the inhaled allergen challenge from baseline divided by the cumulative dose of inhaled cat allergen. PC_20_ was calculated by linear interpolation using the concentrations either side of the 20% fall in FEV1 from baseline (3). The same cat allergen was used for inhalation and PCBAT serum assessment (this differed from the cat allergen used to assess serum collected from Group 1 and 2 subjects). Recovery was monitored using FEV_1_ for 7 hours after final dose of inhaled cat allergen. Early asthmatic response was measured as an area beneath the recovery curve from up to two hours from the last dose of inhaled allergen (EARAUC_0-2hrs_) and the late asthmatic response was measured as an area beneath the curve from three to seven hours from the last dose of inhaled allergen (LARAUC_3-7hrs_); both values we analyzed as natural logarithms to normalize. Participants who had an FEV1 drop of 20% in 0-2 hours and 15% in hours 3-7 were considered dual responders. We then divided the AUC by the cumulative dose of inhaled cat allergen to get a dose response value. No salbutamol was administered throughout this time frame unless the clinician deemed it necessary and the study was then stopped at that point (this only occurred for 1 participant). Spearman’s rank used to compare non normal data and Pearson for normal data.

## Flow cytometry

Cell staining was performed on a 96-well plate using approximately 5x10^5^ cells/well. For PCB characterization, cells were stained with the following antibodies: CD63 (APC), CD123 (Percp-Cy5.5), CD117 (BV605), CD203c (FITC), HLADR (eFluro450) and FcεRI (PE-Cy7) for 20 minutes at 4^o^C.

For PCB characterization, after washing with PBS, cell viability was assessed by staining cells with LIVE/DEAD® Fixable Blue Dead Cell Stain Kit for 10 minutes at 4^o^C. After washing with PBS, cells were stained intracellularly for basophil-specific 2D7 antigen; cells were fixed and permeabilized with intracellular Fixation & Permeabilization buffer set. Fixed cells were incubated in 50μl 2D7 antibody (PE) diluted 1:50 with permeabilization buffer for 1 hour at room temperature. Finally, cells were washed with permeabilization buffer and resuspended in FACS buffer (PBS supplemented with 2% FCS and 2 mM EDTA). PCBs were gated as 2D7^+^/FcεRI^+^/CD117^-^/HLADR^-^ using LSR II (BD Biosciences).

## Fluorescence barcoding

All PCBAT with 16-plex fluorescence barcoding were performed using V-bottomed 96 well plate. Cell stimulation and antibody staining of PCBAT was performed as described in main article’s method section. After cells were stained with antibodies and viability dye, cells were fixed with 1.6% formaldehyde for 10 mins. Then the cells were washed 1x with PBS and centrifuged at 300g for 5 minutes, cells were then permeabilized with methanol containing pacific blue (40, 13.3, 4.43 or 0μg/ml) and Alexafluro 700 (4, 1.33, 0.43 or 0μg/ml) (see Figure E6A). After incubating at room temperature for 15 minutes, cells were washed again with PBS. Cells in individual well were reconstituted in 50µl PBS then pooled, which were then centrifuged at 300g for 5 minutes. Pooled samples were reconstituted in 300µl PBS before flow cytometry analysis.

It is important to arrange the two fluorescent dyes concentrations with specific order, so that the flow cytometry results match the plate plan. For example, for dye A, low concentration to high concentration was arranged from left to right and for dye B low concentration to high concentration was arranged from bottom to top (Figure E6A). When decoding, using dye A to separate the 4 columns then use dye B to separate the 4 wells in each column (Figure E6B). In this study we used CD203c^+^(FITC), FcεRI^+^(PE-Cy7) and CD63 (PE). Pacific blue and Alexafluro 700 as dye A and dye B respectively. The fluorescent dye we used in this paper did not interact with the antibody panel (Figure E7), and can clearly distinguish cells stimulated with different concentrations of allergens, negative control subject (subject A) and responder (subject B) in one pooled sample (Figure E7).

## Immunofluorescence staining

Cells (1-5 x 10^4^ cells) were fixed in 4% paraformaldehyde in PBS at room temperature for 10 minutes and washed once with PBS. Cells were resuspended in 100 μl PBS and left to dry on a slide overnight. Next, cells were incubated in 200 μl diluent (PBS supplemented with 0.1% tween and 10% goat serum) for 30 minutes followed by 1 hour staining in mouse anti-BB1 antibody (1:10). Slides were then rinsed and incubated with Alexa Fluor 555 goat anti-mouse secondary antibody (1:200) for 30 minutes at room temperature. Finally, the slides were mounted with fluoroshield mountant containing DAPI for cell nuclear staining and examined under a Leica DM IL LED microscope using Leica Application Suite software (Leica, UK). Colour overlays were made using ImageJ software (NIH, USA). The primary antibody was omitted as a negative control for every condition tested.

## Calculating EC50 and CDsens

CDmax is the maximal percentage CD63^+^ basophils of a testing subject. High CDmax indicates greater basophil reactivity. EC50 were calculated for subjects with a max PCBAT response >5%. EC50 were determined using non-linear regression, three parameters analysis from GraphPad Prism 7.03 (CA, USA). For PCABT performed with cat allergen extracts, where allergen concentrations were unknown and were presented as dilution factors, an arbitrary value of 1000,000 were used to represent the concentration the neat allergen extracts. For example, allergen extracts diluted with 1:250 were replaced with a value of “4000”. These arbitrary values were used to calculate EC50. For PCBAT performed with peanut allergen extracts, the actual protein concentration was used. CDsens is the reciprocal value of EC50 times 100 (1/EC50 x100). Higher CDsens values indicated greater sensitivity.

**Figure E1**. Gating strategy to remove cell debris, non-singlets and exclusion of dead cells. This is applied to all sample analyses. A representative figure of PCBs cultured at day 16 is shown.

| 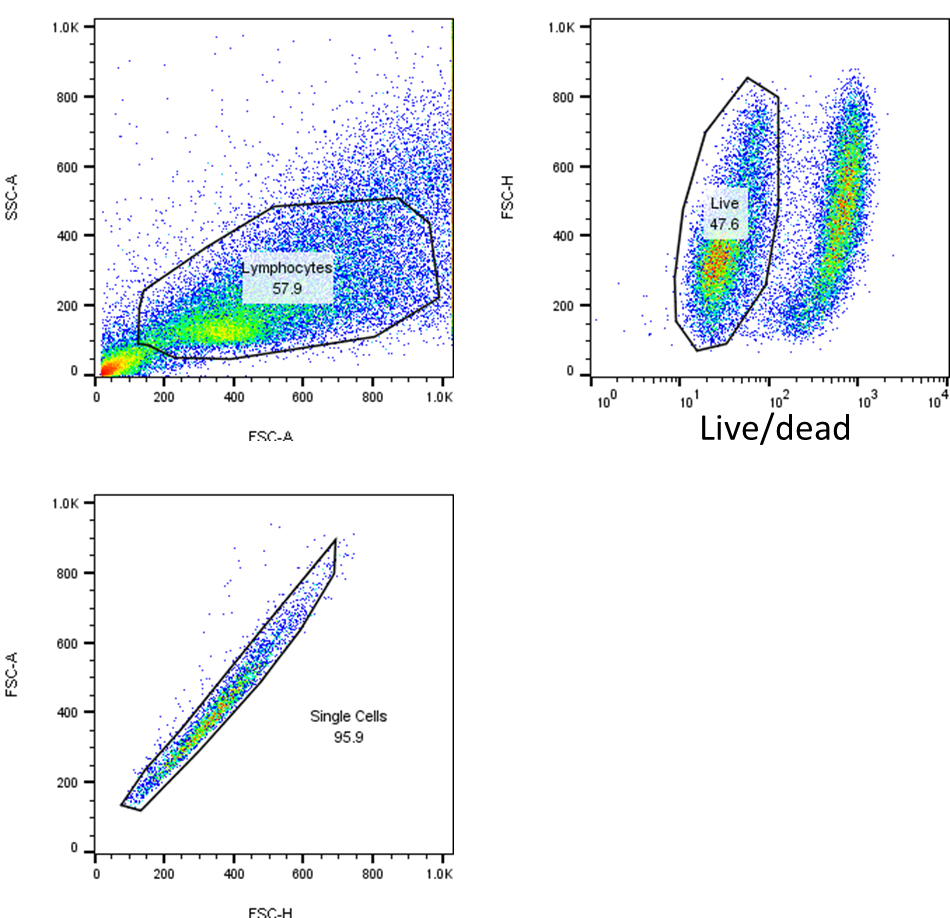 |  |
| --- | --- |
|  |  |

**Figure E2**. Gating strategy to determine the 2D7^+^/FcεRI^+^/HLADR^-^/CD117^-^ population. Fluorescence minus one (FMO) was used to indicate the positive and negative stained cell population. A representative figure of PCBs at day 16 is shown.


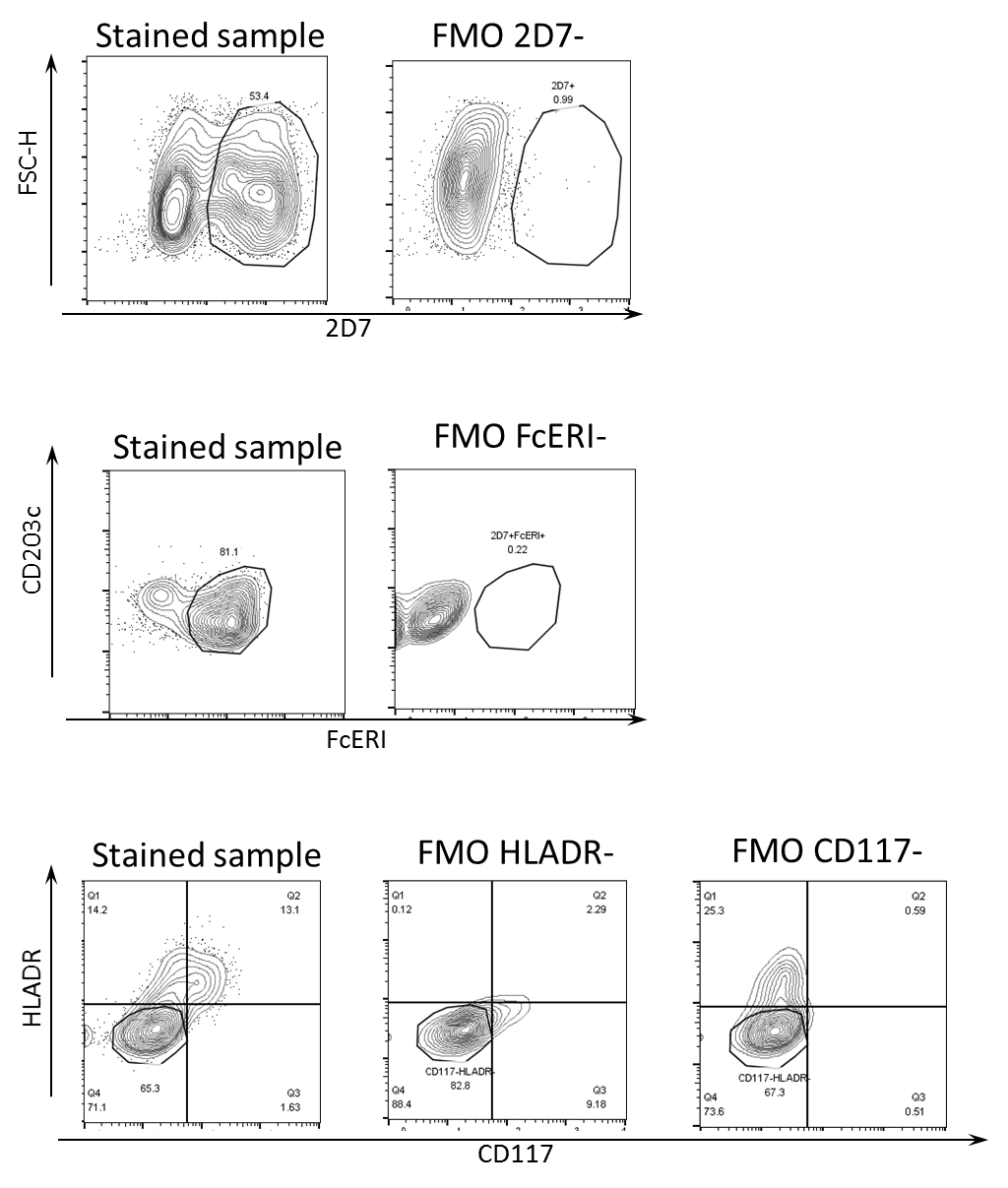


**Figure E3.** Cell morphology characterization using May-Grunwald Giemsa staining on day 16 cells. Cells from day 16 culture (1x10^5^) were centrifuged and fixed with 4% paraformaldehyde at room temperature. Cells were washed with PBS and air-dried on a slide and stained with May-Grunwald for 5 minutes. Slides were washed with PBS for 10 minutes, and then incubated with 1:20 Giemsa solution for 5 minutes. After 2 hours air-dry, the slides were dipped in to xylene. Finally, the slides were mounted with DPX. Images were captured by slide scanner from the University of Manchester bioimaging core facility.


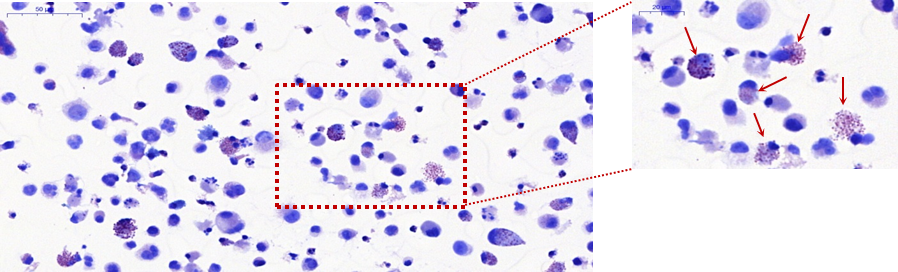


**Figure E4** Characterization of PCBs differentiation during day 7-28 of culture (Donor B)


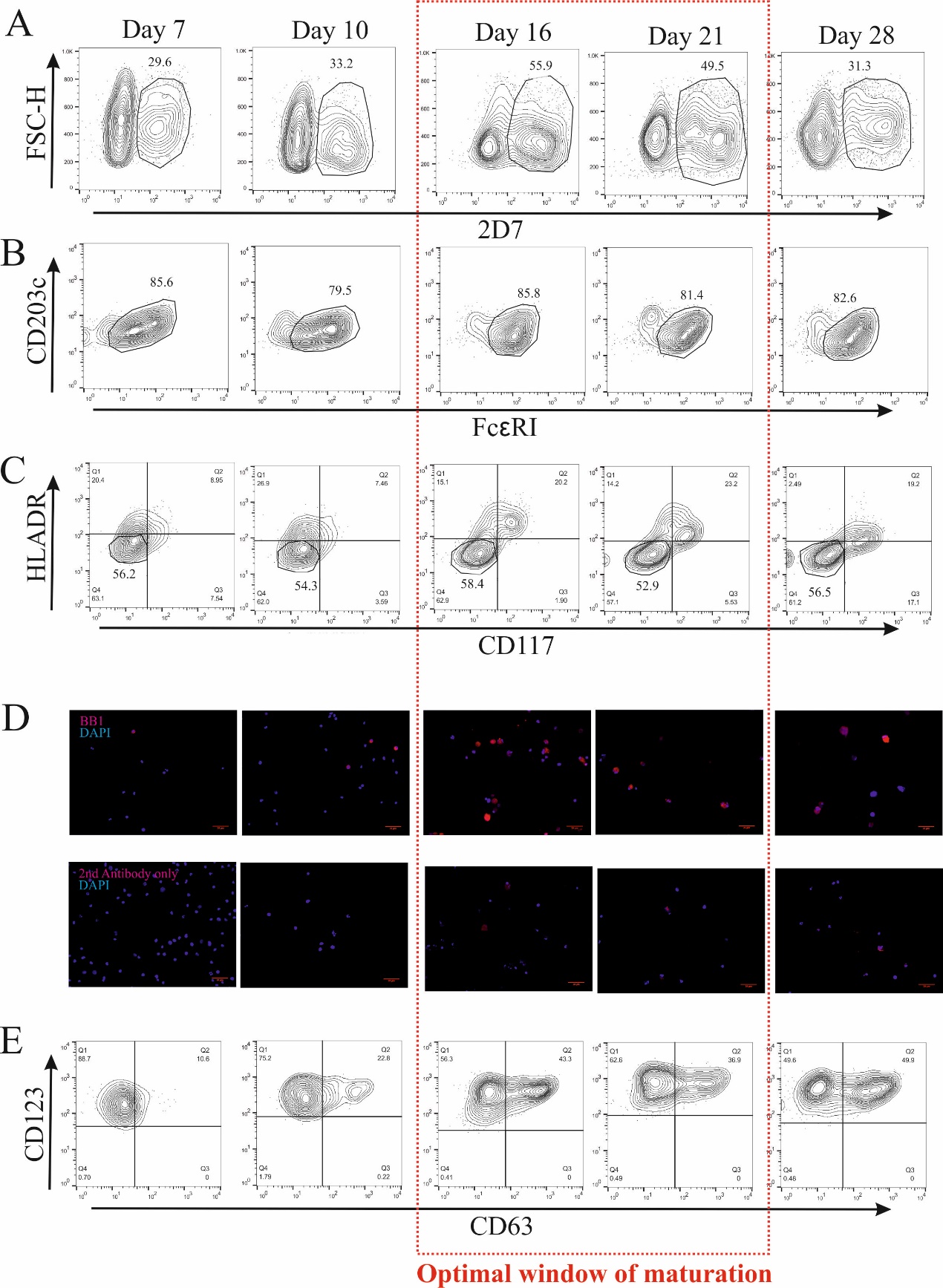


**Figure E5** Examination of passive BAT reproducibility using PCBs. (A) Percentage of PCB activation by anti-IgE stimulation using day 16 basophil culture generated from 5 different donors. (B) Percentage of PCB activation was determined by flow cytometry using CD63 as activation marker. Donor E was used for studying intracellular pERK1/2 expression during anti-IgE stimulation, only 2D7 was used to gate basophil population. (C) The effect of different CD34^+^ cell donors on degranulation performance through culture. CD34^+^ cells were isolated from two donors and cultured in the same condition for 28 days. Cell activity was assessed at day 7, 10, 16, 21 and 28 by sensitizing the cells with IgE and stimulated with anti-IgE. The values are the mean ± range of two technical repeats. D) Representative figures of cell viability at day 7, 10, 16, 21 and 28 days of culture using viability dyes. E) Unstimulated control for subject A and B at day 7, 10, 16, 21 and 28 days of culture .

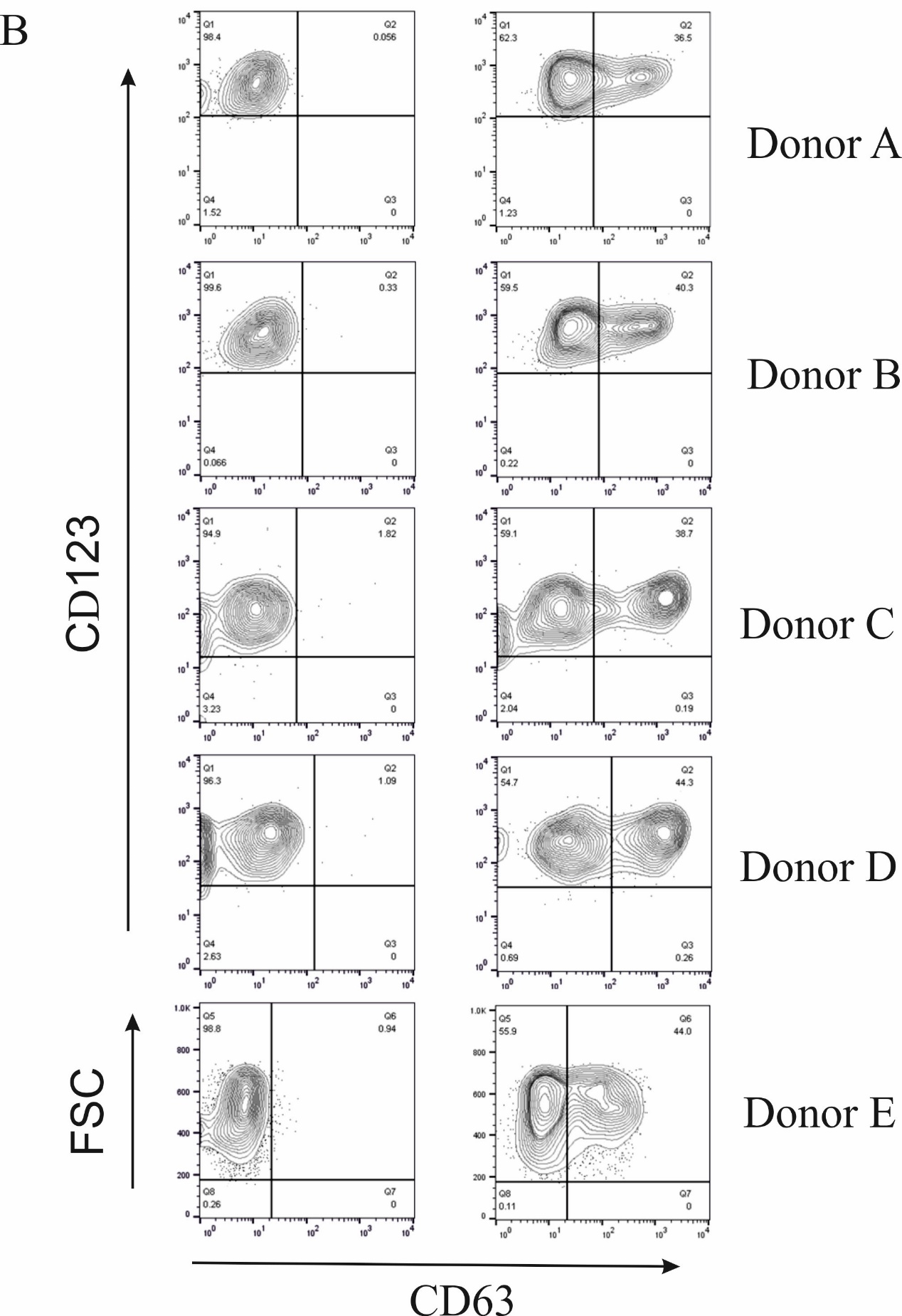

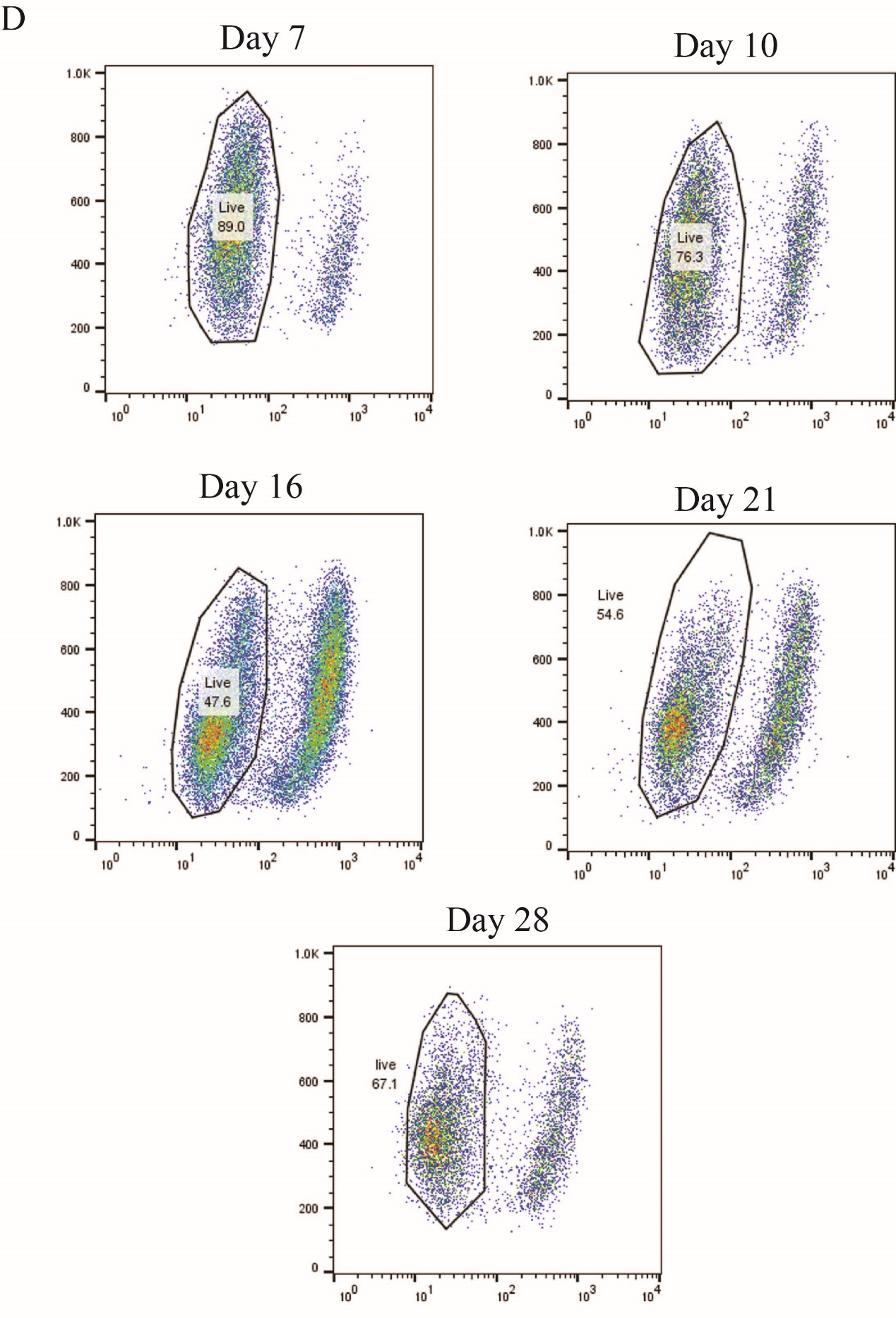


**
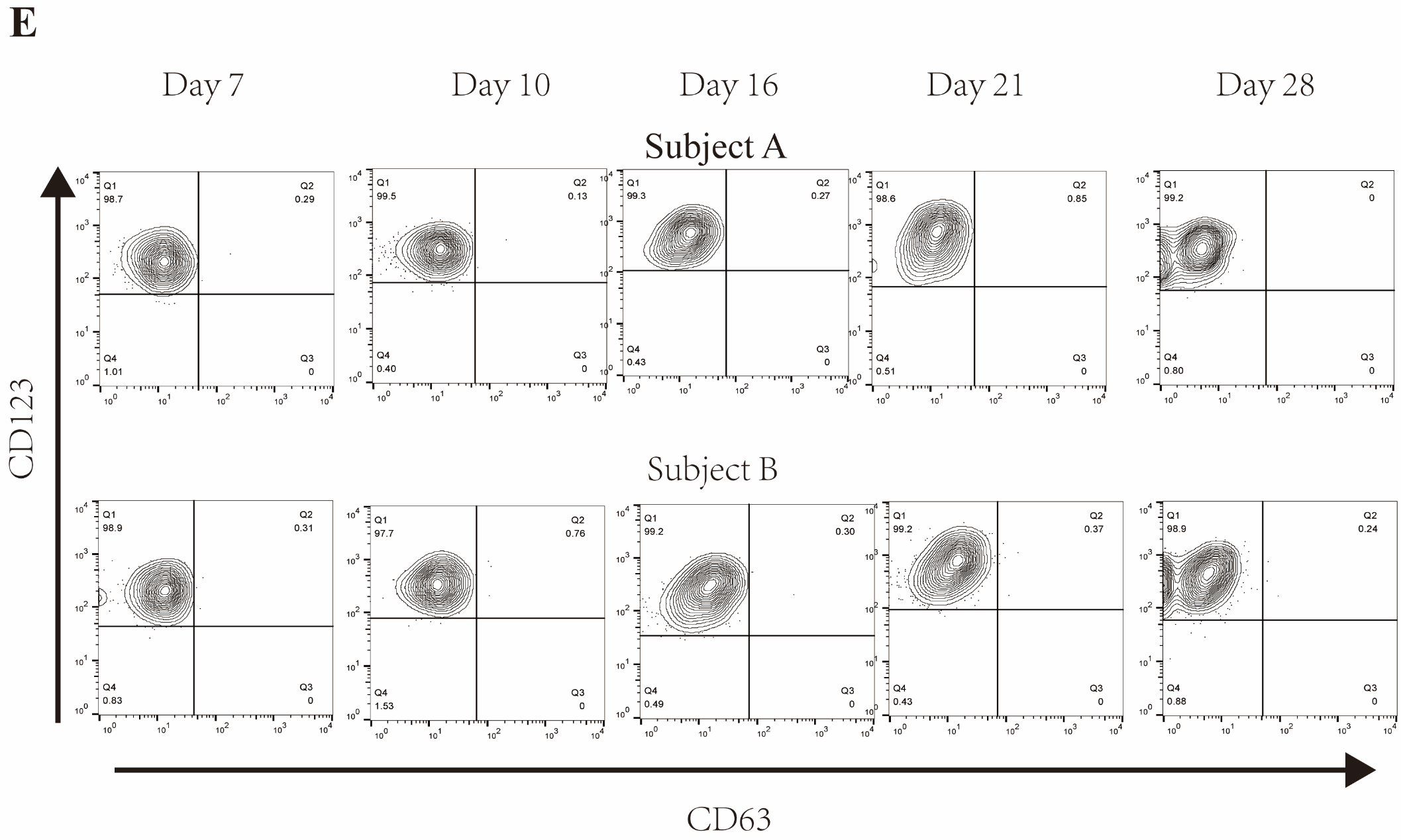
**

**Figure E6 PCBAT with 16-plex fluorescent barcoding**

1. Plate plan for setting up a 16-plex fluorescent barcoded PCBAT on a 96 well plate. Each sample was stained with a different concentration of dye A and dye B before pooled. (B) Illustration of decoding a pooled 16-plex sample using dye A and dye B.

**
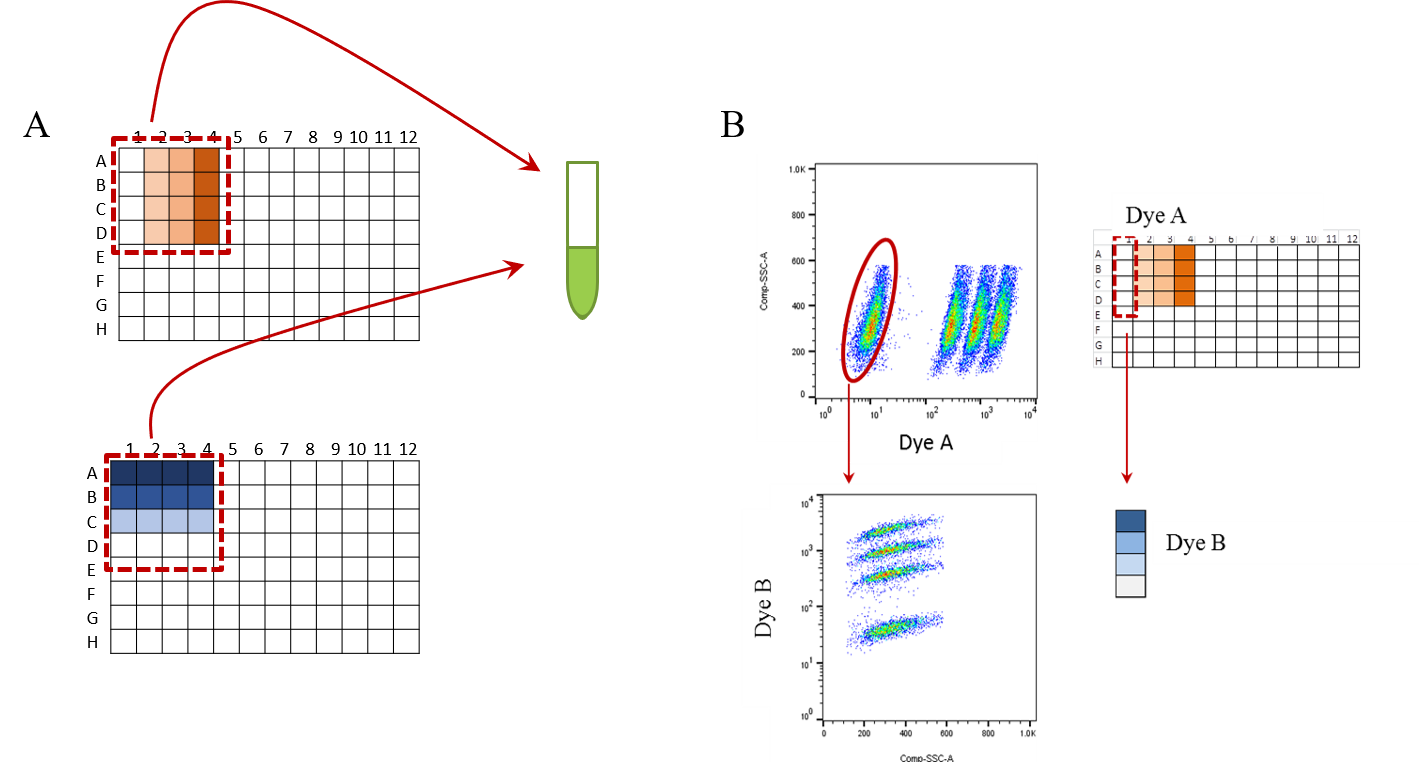
**

**Figure E7 Performance demonstration of PCBAT with 16-plex fluorescent barcoding**

(A) Plate plan for 16-plex fluorescent barcoding with dye A (Pacific blue) and dye B (Alexa Fluor 700) on a 96-well plate format. (B) A representative figure of a 16-plex fluorescent barcoded pooled sample in a PCBAT. Two serum samples were used—subject A was a non-mite sensitizer; subject B was a mite sensitizer. PCBAT were performed using mite allergen with 4 different concentrations, each condition was done in duplicate. After decode the pooled sample with pacific blue (dye A) and Alexafluro700 (dye B), the results demonstrated no interactions between samples and both dyes were not interacting with the antibody panel.

**
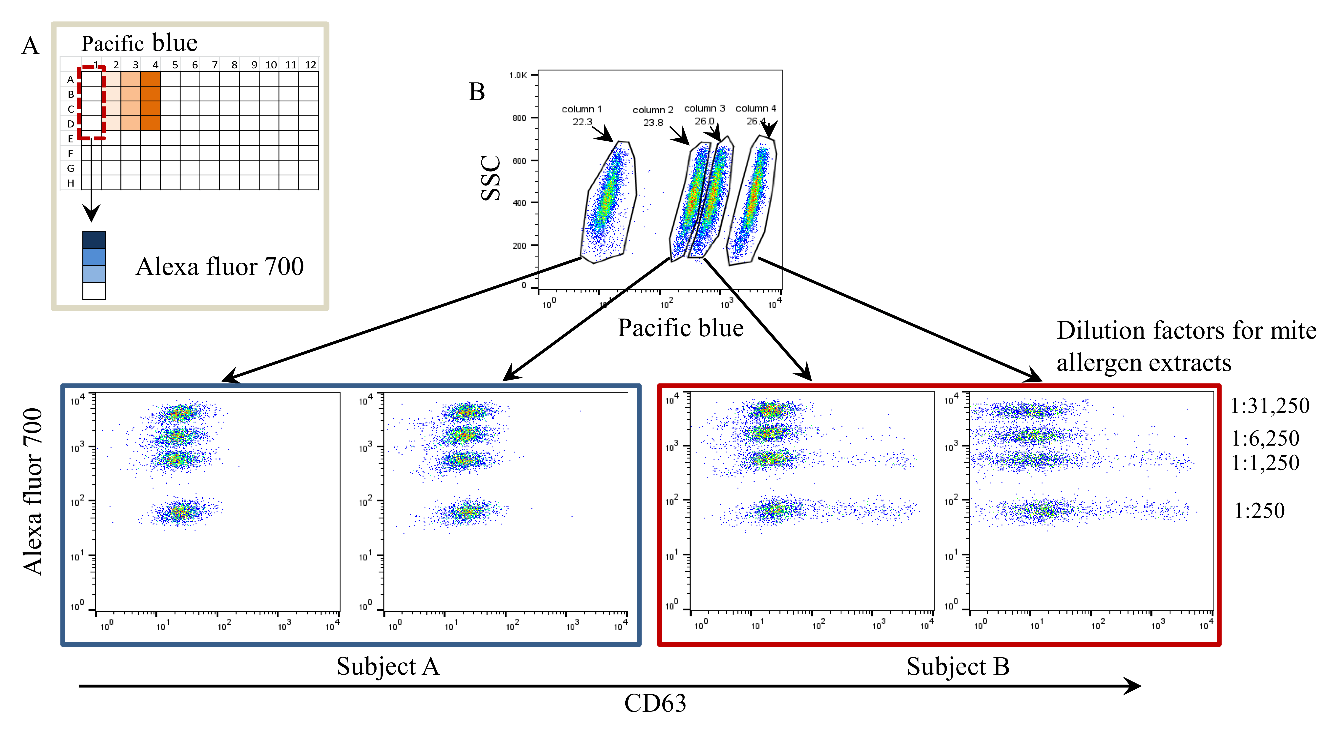
**

**Figure E8** Validating the expression of 2D7 in CD203c positive cells (A) day 16 cells were stained with CD203c (FITC) then sorted for CD203c^+^ cells. (B) Sorted CD203c^+^ cells were stained intracellularly for 2D7.


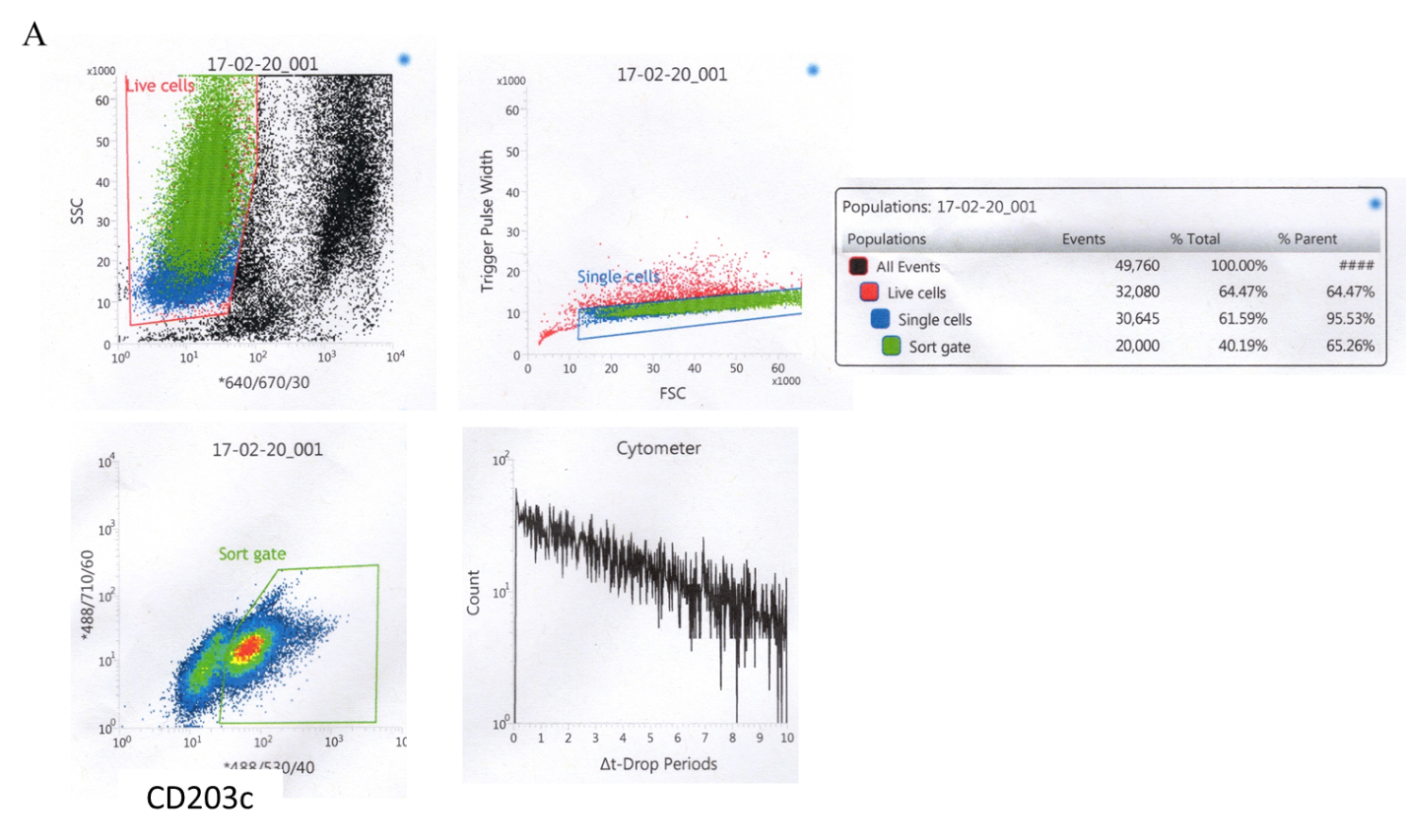


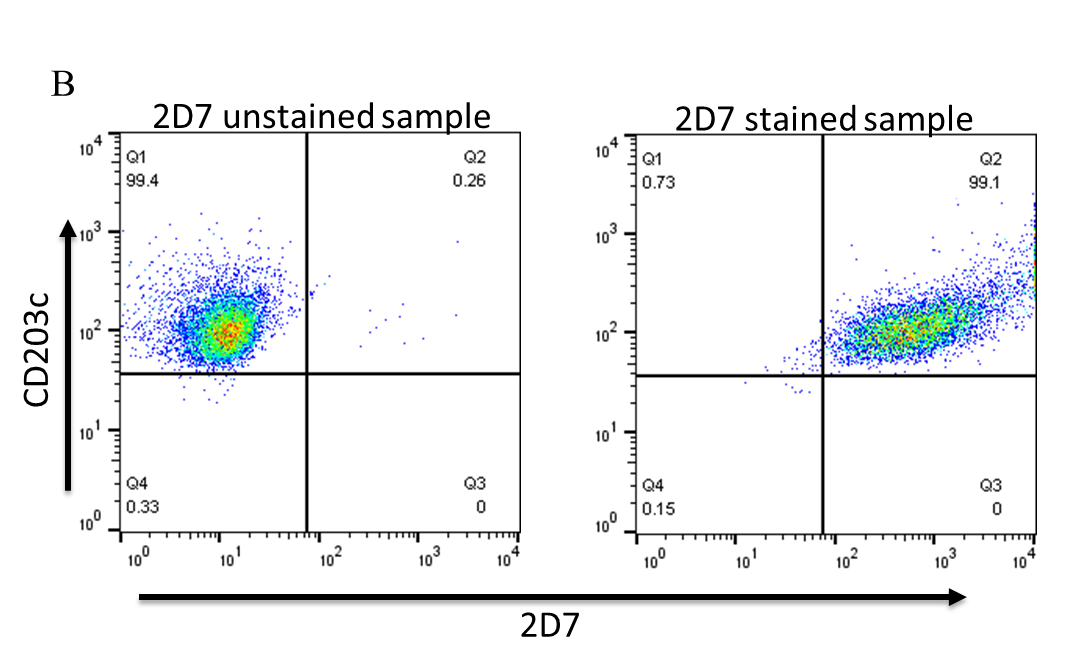


**Figure E9** FcεRI and CD203c positivity and degranulation capability. PCB at day 18 were sensitized with IgE overnight then stimulated with anti-IgE. Cells were stained with FcεRI and CD203c. the degranulation capability of FcεR^+^CD203c^+^, FcεRI^-^CD203c^+^ and FcεRI^-^CD203c^-^ population was evaluated by CD63 expression. Please note that because 2D7 was not used in this panel therefore CD203c^-^ population was also detected. In contrast, in figure E4, after 2D7^+^ pre-selection, only CD203c^+^ population was observed. This is consistent with our sorting data presented in E8.


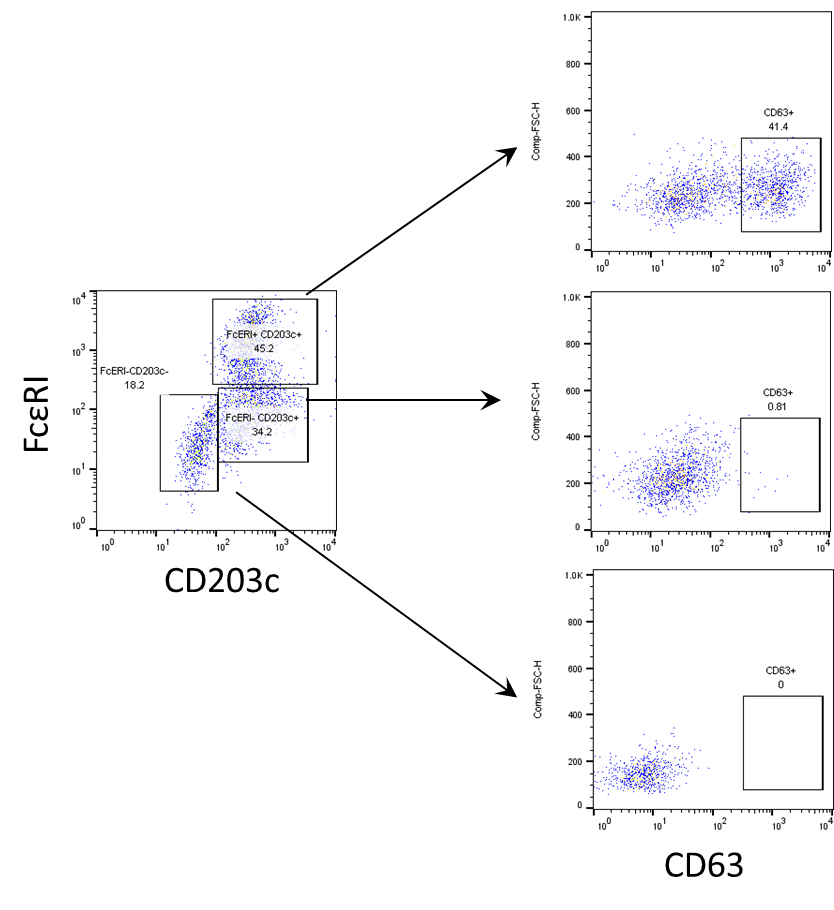


**Figure E10** Group 3 inhaled cat allergen challenge (n=17) (A) the cumulative dose of inhaled cat allergen (in bioequivalent allergen units, BAU) required to cause a drop ≥20% in FEV1. (B) the natural logarithm of the cumulative dose of inhaled cat allergen causing a 20% drop in FEV1 from baseline as calculated with linear interpolation, ln(PC20 cat allergen) for each participant. (C) The correlation between the natural logarithm DRS and PC20, (r = -0.93, p<0.0001). (D) No difference in mean PCABAT AUC was seen comparing dual responders to those with only an early response to inhaled cat allergen, (p = 0.65). (E) ln(EARAUC_0-2hrs_) had a weak correlation with PCBAT AUC, (r = 0.51, p = 0.038). (F) ln(LARAUC_3-7hrs_) had no correlation with PCBAT AUC, (r = 0.27, p = 0.29). (G) no correlation seen between titrated skin test reactivity and ln(PC20 cat allergen) (r = -0.35, p=0.17). (H) There a significant correlation between the ln(PC_20_ cat allergen/ PC_20_ methacholine) and PCBAT AUC, (r = 0.51, p = 0.035).

**
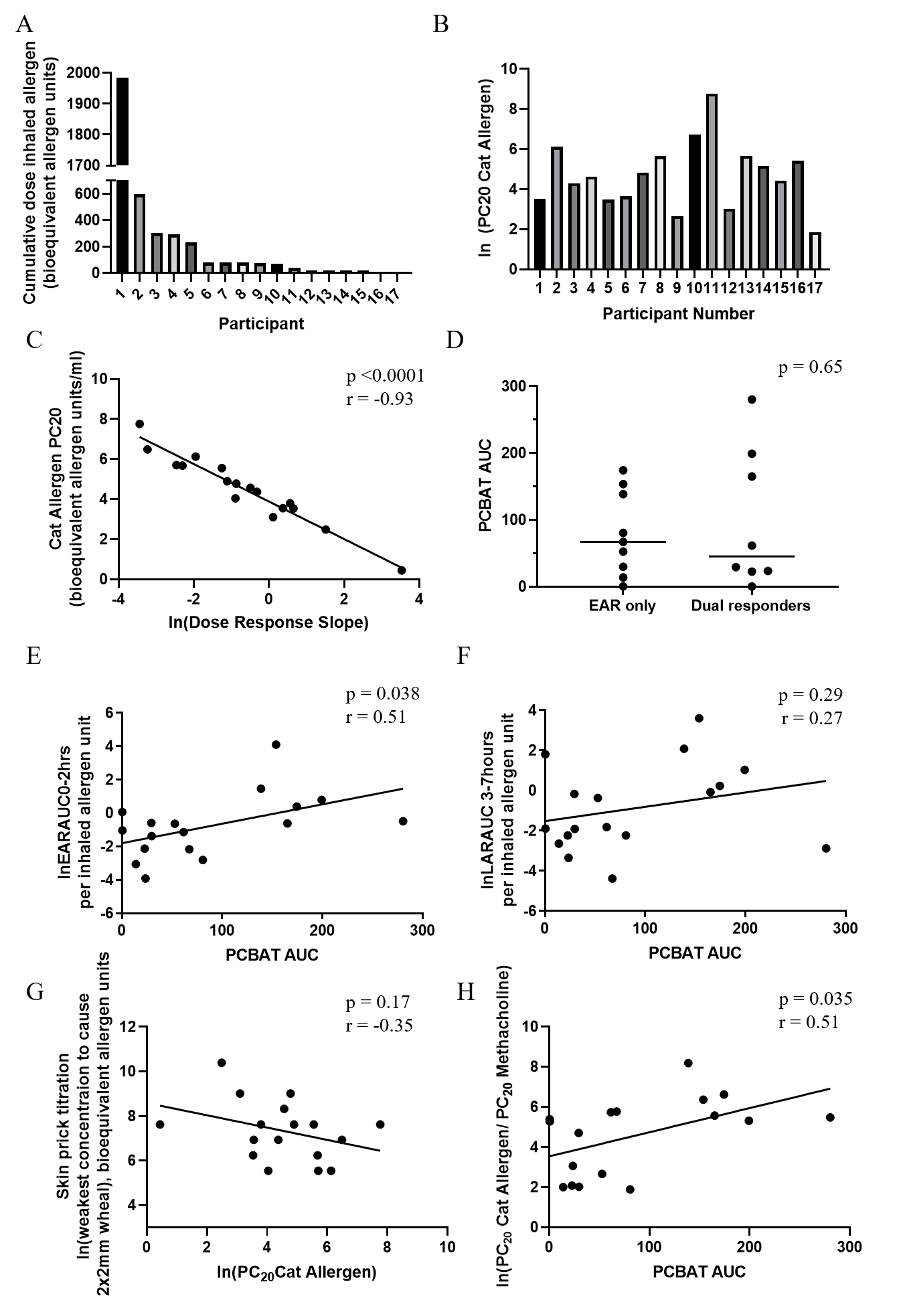
**

**Figure E11** The relationship between AUC of PCBAT from the 30 peanut allergic subjects (Group 4) and levels of (A) Ara h 3, (B) Ara h 8 and (C) Ara h 9 sIgE (ImmunoCap). (D) The relationship between AUC of PCBAT from the 15-challenge proven peanut allergic subjects and levels of Ara h 6 sIgE (ISAC). P value and R square values were calculated using spearman test.

**Figure E12** levels of Fel d 1 and Can f 1 in cat allergen extracts. Cat allergen samples from ALK and Jubilant HollisterStier were tested for the levels of Fel d 1 and presence of dog allergen components using Fel d 1 and Can f 1 ELISA kits respectively.

**Table E1.** Summary of PCB activation tests (% of CD63 positive cells) using cat allergen extract in Group 1. Subjects were sensitized to cat allergen extracts, anti-IgE were used as positive control. Whole cat sIgE were also listed. Cat sIgE negative subjects were given a value of “0.3”, cat sIgE >100KU/L were given a value of “101”.

|  | Cat | | | | | | | | |  |  |  |  |  |
| --- | --- | --- | --- | --- | --- | --- | --- | --- | --- | --- | --- | --- | --- | --- |
| subject ID | Ctrl | 1:781,250 | 1:156,250 | 1:31,250 | 1:6,250 | 1:1,250 | 1:250 | Anti-IgE | Cat sIgE | Total IgE (KU/L) | AUC | EC50 | CDsens | CDmax |
| 1 | 0.36 | 0.26 | 0.19 | 0.42 | 0.45 | 0.73 | 0.46 | 37.28 | 0.50 | 11.8 | 2.45 | NR | NR | NR |
| 2 | 1.19 | 0.86 | 1.23 | 1.97 | 0.76 | 2.38 | 6.34 | 51.48 | 0.50 | 140 | 10.97 | 977.89 | 0.10 | 6.34 |
| 3 | 0.79 | 0.60 | 1.69 | 6.30 | 6.00 | 4.02 | 8.70 | 49.00 | 0.50 | 240 | 23.35 | 8.48 | 11.79 | 8.70 |
| 4 | 0.23 | 0.07 | 0.13 | 0.65 | 6.39 | 13.65 | 15.90 | 39.77 | 0.50 | 70 | 28.95 | 220.5 | 0.45 | 15.90 |
| 5 | 1.27 | 1.85 | 1.88 | 5.33 | 6.39 | 4.02 | 2.59 | 47.72 | 0.60 | 70 | 21.39 | 18.96 | 5.27 | 6.39 |
| 6 | 1.60 | 0.90 | 0.44 | 0.28 | 1.75 | 13.83 | 23.47 | 41.63 | 0.60 | 110 | 29.72 | 775.5 | 0.13 | 23.47 |
| 7 | 0.61 | 0.94 | 0.57 | 0.59 | 3.91 | 9.34 | 15.35 | 45.05 | 0.90 | 65.2 | 23.32 | 782.3 | 0.13 | 15.35 |
| 8 | 1.22 | 1.33 | 1.29 | 3.45 | 2.52 | 5.41 | 2.20 | 47.65 | 1.10 | 100 | 15.69 | 12.44 | 8.04 | 5.41 |
| 9 | 1.11 | 10.09 | 14.56 | 26.00 | 18.95 | 12.45 | 9.11 | 47.42 | 1.33 | 120 | 87.15 | 2.37 | 42.19 | 26.00 |
| 10 | 0.55 | 0.40 | 0.32 | 0.40 | 1.10 | 12.27 | 28.90 | 47.48 | 2.10 | 1000 | 29.21 | 984.11 | 0.10 | 28.90 |
| 11 | 0.50 | 0.37 | 0.56 | 1.42 | 8.42 | 20.50 | 21.55 | 41.03 | 2.20 | 27.2 | 42.28 | 207.44 | 0.48 | 21.55 |
| 12 | 0.68 | 15.55 | 29.25 | 23.55 | 23.65 | 25.60 | 21.60 | 47.88 | 3.60 | 130 | 128.74 | 1.256 | 79.62 | 29.25 |
| 13 | 1.36 | 0.59 | 0.52 | 0.48 | 0.90 | 4.29 | 10.06 | 54.22 | 7.70 | 57.8 | 12.48 | 1170.7 | 0.09 | 10.06 |
| 14 | 0.86 | 5.08 | 15.00 | 29.10 | 35.10 | 36.30 | 37.65 | 51.50 | 8.50 | 110 | 139.83 | 9.79 | 10.21 | 37.65 |
| 15 | 0.52 | 11.95 | 24.25 | 28.65 | 29.70 | 29.95 | 24.60 | 39.82 | 25.90 | 190 | 137.06 | 1.67 | 59.88 | 29.95 |
| 16 | 1.60 | 13.54 | 15.95 | 24.70 | 30.30 | 25.35 | 21.25 | 32.32 | 28.30 | 970 | 121.26 | 1.89 | 52.91 | 30.30 |
| 17 | 0.45 | 15.55 | 24.80 | 29.00 | 26.05 | 21.00 | 23.55 | 21.23 | 32.10 | 2400 | 128.40 | 1.08 | 92.59 | 29.00 |
| 18 | 0.31 | 1.17 | 8.01 | 10.45 | 12.20 | 10.25 | 9.47 | 16.07 | 101.0 | 2001 | 46.96 | 3.89 | 25.71 | 12.20 |
| Ctrl 1 | 0.79 | 0.48 | 0.96 | 0.62 | 1.27 | 0.43 | 0.69 | 50.23 | 0.30 | 170 | 4.49 | NA | NA | NA |
| Ctrl 2 | 0.71 | 0.15 | 0.40 | 0.42 | 0.61 | 0.95 | 0.80 | 59.07 | 0.30 | 7 | 3.27 | NA | NA | NA |
| Ctrl 3 | 0.75 | 0.91 | 0.88 | 1.57 | 0.51 | 1.20 | 2.49 | 46.92 | 0.30 | 45.2 | 6.68 | NA | NA | NA |
| Ctrl 4 | 0.40 | 0.36 | 0.34 | 0.40 | 2.00 | 2.68 | 0.59 | 47.42 | 0.30 | 140 | 6.25 | NA | NA | NA |
| Ctrl 5 | 1.64 | 0.60 | 0.74 | 2.26 | 0.90 | 1.07 | 1.61 | 45.35 | 0.30 | 150 | 7.17 | NA | NA | NA |
| Ctrl 6 | 0.44 | 0.80 | 0.03 | 0.19 | 0.33 | 0.63 | 9.78 | 25.85 | 0.30 | 4900 | 7.08 | 1846.95 | 0.05 | 9.78 |

NA = not available, NR = PCBAT non responder

**Table E2.** Summary of PCB activation tests (%CD63+ cells) using cat allergen extracts on the four subjects treated with omalizumab (Group 2). Whole cat sIgE were also listed. Specific IgE negative subjects were given a value of “0.3”, sIgE >100KU/L were given a value of “101”.

|  | Cat allergen extracts (dilution factors) | | | | | | |  |  |  |
| --- | --- | --- | --- | --- | --- | --- | --- | --- | --- | --- |
| subject ID | Ctrl | 1:781,250 | 1:156,250 | 1:31,250 | 1:6,250 | 1:1,250 | 1:250 | Total IgE (KU/L) | Cat sIgE (KU/L) | anti-IgE (KU/L) |
| 1 | 0.38 | 0.81 | 0.16 | 0.23 | 0.28 | 0.51 | 0.67 | 550 | 0.80 | 26.78 |
| 2 | 0.38 | 0.18 | 0.31 | 0.37 | 0.31 | 0.26 | 0.80 | 66.3 | 101.00 | 29.85 |
| 3 | 0.23 | 0.46 | 0.40 | 0.24 | 0.19 | 0.12 | 0.47 | 890 | 0.30 | 32.05 |
| 4 | 1.50 | 0.73 | 0.28 | 0.68 | 0.49 | 1.52 | 1.67 | 720 | 42.10 | 33.40 |

**Table E3.** Summary of PCB activation tests (% of CD63 positive cells) using cat allergen extract in Group 3. Subjects clinically validated cat allergic using inhalant cat allergen challenge, anti-IgE were used as positive control.

|  | Dilution factor for cat allergen in PCBAT | | | | | | | Positive  Control |  |  |  | Inhaled cat allergen challenge | | Cat pelt Skin Prick Test |
| --- | --- | --- | --- | --- | --- | --- | --- | --- | --- | --- | --- | --- | --- | --- |
|  | 0 | 1:781250 | 1:156250 | 1:31250 | 1:6250 | 1:1250 | 1:250 | Anti-IgE | AUC | Cdsense | Cdmax | PC20 Cat Allergen | Dose response slope (DRS) | Minimum concentration of allergen resulting in a 2x2 mm wheal (BAU) |
| 1 | 0.12 | 0.385 | 0.18 | 0.505 | 1.005 | 7.36 | 28.1 | 40.45 | 23.55 | 4.56 | 28.1 | 6.42 | 0.01 | 4.88 |
| 2 | 0.515 | 0.185 | 0.15 | 0.195 | 1.37 | 4.99 | 13.68 | 27.35 | 13.99 | 3.75 | 13.68 | 14.15 | 0.04 | 9.76 |
| 3 | 0.395 | 0.295 | 0.235 | 0.49 | 2.04 | 8.965 | 20.95 | 25.4 | 22.7 | 6.75 | 20.95 | 33.12 | 0.098 | 19.49 |
| 4 | 1.025 | 1.32 | 6.085 | 14.505 | 13.7 | 27.45 | 34.2 | 21.45 | 80.67 | 15.85 | 34.2 | 20.70 | 0.07 | 38.91 |
| 5 | 0.85 | 0.29 | 1 | 3.75 | 6.63 | 12.2 | 11.06 | 31.15 | 29.83 | 92.17 | 12.2 | 32.46 | 0.26 | 38.91 |
| 6 | 0.08 | 0.31 | 0.27 | 0 | 0.185 | 0.93 | 1.495 | 39.9 | 0.62 | NR | NR | 83.10 | 0.34 | 1.22 |
| 7 | 0.31 | 0.195 | 0.965 | 7.495 | 8.905 | 29.1 | 40.75 | 18.9 | 67.19 | 18.17 | 40.75 | 73.70 | 0.29 | 4.88 |
| 8 | 0.56 | 2.53 | 12.75 | 35.65 | 35.8 | 50.7 | 54.55 | 48.05 | 165 | 409.33 | 54.55 | 102.51 | 0.31 | 38.91 |
| 9 | 0.57 | 0.22 | 1.485 | 6.8 | 9.56 | 21.35 | 26.05 | 46.4 | 52.73 | 29.44 | 26.05 | 38.09 | 0.25 | 4.88 |
| 10 | 1.095 | 0.565 | 1.94 | 6.445 | 4.17 | 7.89 | 15.7 | 19.55 | 29.41 | 0.29 | 15.7 | 174.16 | 0.68 | 38.91 |
| 11 | 0.7 | 0.37 | 1.565 | 7.925 | 8.15 | 27.4 | 31.6 | 30.3 | 61.56 | 30.03 | 31.6 | 125.21 | 0.5 | 9.76 |
| 12 | 0.525 | 3.425 | 16.8 | 38.55 | 41.5 | 66.75 | 63.9 | 39.3 | 199.2 | 287.6 | 66.75 | 450.34 | 1.65 | 1.22 |
| 13 | 0.495 | 1.28 | 7.065 | 28.1 | 43.1 | 64.55 | 60.05 | 45.95 | 174.4 | 198.26 | 64.55 | 284.29 | 1.09 | 9.76 |
| 14 | 0.265 | 0.325 | 0 | 0.19 | 0.775 | 0.16 | 0.205 | 6.925 | 0.4575 | NR | NR | 223.63 | 0.92 | 4.88 |
| 15 | 1.47 | 16.35 | 51.2 | 51.5 | 54.2 | 73.7 | 65.4 | 32.2 | 280.4 | 3676.47 | 73.7 | 290.03 | 1.22 | 19.49 |
| 16 | 1.29 | 1.18 | 6.68 | 18.65 | 31.7 | 52.25 | 55.15 | 47.2 | 138.7 | 89.5 | 55.15 | 828.82 | 3.17 | 0.31 |
| 17 | 0.69 | 2.65 | 12.35 | 28.9 | 33.4 | 48.55 | 55.05 | 37.4 | 153.7 | 162.26 | 55.05 | 6438.17 | 43.5 | 4.88 |

NR –PCBAT non responder

**Table E4** Skin prick test results for Group 3 patients Skin prick wheal size results after 10 minutes, length x width in millimeters.

| new | Dog | Cat pelt | horse | feather | dictyoptera | HDMdf | HDMdp | alternaria | hormodendrum | aspergillus | tree | grass mix |
| --- | --- | --- | --- | --- | --- | --- | --- | --- | --- | --- | --- | --- |
| 1 | 3x3 | 9x9 | 0x0 | 0x0 | 0x0 | 2x2 | 2x2 | 0x0 | 0x0 | 0x0 | 0x0 | 3x3 |
| 2 | 0x0 | 4x4 | 2x2 | 0x0 | 0x0 | 3x3 | 3x3 | 2x2 | 1x1 | 1x2 | 2x2 | 3x3 |
| 3 | 2x2 | 4x4 | 0x0 | 0x0 | 0x0 | 6x6 | 6x6 | 0x0 | 0x0 | 0x0 | 0x0 | 1x1 |
| 4 | 2x2 | 3x3 | 5x5 | 0x0 | 0x0 | 2x2 | 2x2 | 2x2 | 0x0 | 0x0 | 0x0 | 5x5 |
| 5 | NA | | | | | | | | | | | |
| 6 | 0x0 | 2x2 | 0x0 | 0x0 | 2x2 | 0x0 | 0x0 | 0x0 | 0x0 | 0x0 | 3x3 | 5x5 |
| 7 | 3x3 | 6x6 | 10x11 | 0x0 | 0x0 | 0x0 | 0x0 | 0x0 | 0x0 | 0x0 | 0x0 | 0x0 |
| 8 | 5x5 | 10x10 | 11x8 | 0x0 | 0x0 | 7x6 | 9x4 | 3x3 | 7x7 | 3x3 | 10x10 | 7x4 |
| 9 | 4x4 | 6x6 | 0x0 | 4x4 | 0x0 | 7x7 | 6x6 | 1x2 | 2x2 | 2x2 | 2x2 | 4x4 |
| 10 | 3x3 | 6x5 | 6x4 | 0x0 | 2x2 | 2x2 | 0x0 | 0x0 | 2x2 | 0x0 | 0x0 | 0x0 |
| 11 | 0x0 | 5x5 | 5x5 | 0x0 | 0x0 | 6x6 | 6x6 | 0x0 | 0x0 | 0x0 | 0x0 | 9x9 |
| 12 | 3x3 | 8x8 | 5x5 | 0x0 | 0x0 | 0x0 | 0x0 | 10x10 | 5x5 | 0x0 | 0x0 | 0x0 |
| 13 | 0x0 | 6x6 | 0x0 | 0x0 | 0x0 | 0x0 | 0x0 | 0x0 | 0x0 | 0x0 | 0x0 | 0x0 |
| 14 | 2x2 | 2x2 | 0x0 | 0x0 | 0x0 | 4x4 | 4x4 | 1x1 | 2x2 | 2x2 | 2x2 | 3x3 |
| 15 | 0x0 | 6x6 | 5x5 | 0x0 | 0x0 | 0x0 | 0x0 | 0x0 | 0x0 | 0x0 | 0x0 | 3x3 |
| 16 | 0x0 | 8x10 | 2x2 | 0x0 | 0x0 | 5x5 | 5x5 | 4x4 | 0x0 | 0x0 | 9x9 | 10x10 |
| 17 | 3x3 | 7x6 | 0x0 | 0x0 | 0x0 | 10x10 | 9x9 | 0x0 | 0x0 | 0x0 | 0x0 | 2x2 |

NA: data not available

**Table E5.** Summary of PCB activation tests and sIgE measure for 30 physician diagnosed peanut allergic subject and 4 peanut non-sensitized controls (Group 4). Subjects 1-15 were challenge proven peanut allergic subjects.

|  | Peanut concentration in PCBAT (ng/ml) | | | | |  | Peanut specific IgE (ISU) | | | | | | Total IgE(KU/L) |  |  |  |  |
| --- | --- | --- | --- | --- | --- | --- | --- | --- | --- | --- | --- | --- | --- | --- | --- | --- | --- |
|  | ctrl | 1 | 10 | 100 | 1000 | anti-IgE | whole peanut | Ara h1 | Ara h2 | Ara h3 | Ara h8 | Ara h9 |  | AUC | EC50 | CDsens | CDmax |
| 1 | 0.65 | 0.87 | 5.52 | 21.55 | 35.4 | 18.25 | 15.03 | 0.3 | 13.15 | 0.3 | 1.3 | 0.3 | 370.0 | 45.97 | 77.39 | 1.29 | 35.4 |
| 2 | 0.36 | 1 | 1.37 | 11.3 | 20.3 | 7.3 | 54.51 | 2.47 | 28.55 | 0.76 | 43.92 | 1.51 | NA | 23.95 | 104.1 | 0.96 | 20.2 |
| 3 | 1.13 | 9.87 | 19.55 | 46.05 | 63.7 | 24.35 | 101 | 44.74 | 38.65 | 22.91 | 0.3 | 0.3 | NA | 107.89 | 52.84 | 1.89 | 63.7 |
| 4 | 2.1 | 1.78 | 2.4 | 5.78 | 15.7 | 11.44 | 10.12 | 6.55 | 2.2 | 0.3 | 3.07 | 1.83 | NA | 18.86 | 382 | 0.26 | 15.7 |
| 5 | 1.07 | 0.99 | 1.09 | 1.66 | 5.73 | 16.9 | 8.57 | 0.62 | 2.52 | 2.91 | 9.3 | 0.3 | 190.0 | 7.14 | 2161 | 0.05 | 5.73 |
| 6 | 0.97 | 1.34 | 8.34 | 27.55 | 55.6 | 5.17 | 101 | 1.81 | 85.31 | 1.53 | 0.3 | 0.3 | NA | 65.52 | 134.8 | 0.74 | 55.6 |
| 7 | 1.92 | 1.75 | 1.34 | 8.63 | 23.3 | 37.4 | 2.33 | 1.76 | 0.86 | 0.3 | 0.3 | 0.3 | NA | 24.33 | 283.3 | 0.35 | 23.3 |
| 8 | 1.66 | 4.42 | 17.55 | 38.45 | 68 | 23.45 | 65.84 | 19.57 | 39.53 | 7.97 | 6.54 | 0.3 | 150.0 | 95.25 | 104.2 | 0.96 | 68 |
| 9 | 1.08 | 2.42 | 5.19 | 14.4 | 22.85 | 22.05 | 9.09 | 0.3 | 3.74 | 0.3 | 13.33 | 0.3 | 1400.0 | 33.98 | 81.93 | 1.22 | 22.85 |
| 10 | 0.69 | 1.16 | 2.2 | 14.25 | 23.15 | 30.1 | 3.37 | 2.63 | 0.68 | 0.3 | 0.3 | 0.3 | 110.0 | 29.53 | 81.91 | 1.22 | 23.15 |
| 11 | 1.48 | 7.54 | 26.8 | 52.45 | 73.4 | 20.35 | 101 | 101 | 101 | 20.75 | 0.3 | 0.3 | NA | 124.23 | 36.63 | 2.73 | 73.4 |
| 12 | 1.98 | 1.15 | 2.49 | 19.05 | 24.05 | 14 | 12.66 | 5.89 | 2.85 | 1.26 | 0.3 | 0.3 | NA | 35.71 | 43.82 | 2.28 | 24.05 |
| 13 | 1 | 1.09 | 2.33 | 6.75 | 4.99 | 27.8 | 0.62 | 0.3 | 0.32 | 0.3 | 0.3 | 0.3 | NA | 13.17 | 13.16 | 7.60 | 6.75 |
| 14 | 0.75 | 0.64 | 1.08 | 1.15 | 5.3 | 25.55 | 0.59 | 0.3 | 0.11 | 0.3 | 0.3 | 0.3 | NA | 5.90 | 194.87 | 0.51 | 5.3 |
| 15 | 0.63 | 0.9 | 0.85 | 9.32 | 19.15 | 30.35 | 3.94 | 2.35 | 1.76 | 0.3 | 5.7 | 0.3 | NA | 20.96 | 142.2 | 0.70 | 19.15 |
| 16 | 0.94 | 1.22 | 14.4 | 36.35 | 59.05 | 23.75 | 3.94 | 11 | 17 | 7.6 | 0.4 | 0.3 | NA | 81.97 | 68.88 | 1.45 | 59.05 |
| 17 | 0.71 | 3.09 | 9.79 | 36.65 | 30.35 | 22.45 | NA | 2 | 17 | 0.3 | NA | NA | 640.0 | 65.01 | 16.53 | 6.05 | 36.65 |
| 18 | 1.05 | 1.49 | 8.77 | 27.9 | 33.15 | 26.2 | 24 | 2.1 | 15 | 1.2 | 0.3 | 0.3 | 430 | 55.26 | 26.65 | 3.75 | 33.15 |
| 19 | 0.76 | 0.97 | 6.96 | 22.8 | 25.45 | 22.3 | NA | NA | 11.8 | NA | 0.3 | 0.3 | 46.5 | 43.84 | 22.85 | 4.38 | 25.45 |
| 20 | 1.04 | 1.27 | 2.25 | 5.45 | 12.95 | 8.92 | 17.1 | 76.9 | 28.2 | 0.3 | 0.3 | NA | 4100 | 15.97 | 256 | 0.39 | 12.95 |
| 21 | 1.13 | 3 | 16.15 | 39.05 | 57.15 | 23 | NA | 20.6 | 35.1 | 2.8 | 0.3 | 0.3 | 630 | 87.34 | 48.06 | 2.08 | 57.15 |
| 22 | 1.66 | 3.23 | 16.7 | 47.9 | 66.15 | 15.3 | 52.8 | 36 | 17 | 30 | 0.3 | 0.3 | NA | 101.74 | 41.57 | 2.41 | 66.15 |
| 23 | 0.48 | 3.57 | 20.8 | 43.6 | 66.65 | 23.05 | NA | 21.6 | 101 | 22.2 | 3.1 | 6.5 | 960 | 101.54 | 54.14 | 1.85 | 66.65 |
| 24 | 1.99 | 1.62 | 1.32 | 10.7 | 15 | 14.98 | 101 | 0.3 | 12.5 | NA | NA | NA | 3200 | 22.14 | 63.84 | 1.57 | 15 |
| 25 | 0.82 | 2.59 | 12.55 | 50.35 | 55.35 | 13.25 | 11.8 | 14 | 61 | 21 | 1 | 0.3 | 1900 | 93.48 | 26.08 | 3.83 | 55.35 |
| 26 | 2.49 | 5.33 | 20.1 | 51.8 | 69.05 | 20.9 | NA | 68 | 59.1 | 34.8 | 1 | 0.3 | 780 | 113.00 | 36.43 | 2.74 | 69.05 |
| 27 | 0.71 | 1.58 | 13.8 | 39.5 | 45.65 | 31.15 | 101 | 4.4 | 49.2 | 3.4 | 0.3 | 0.3 | 980 | 78.06 | 21.64 | 4.62 | 45.65 |
| 28 | 1.94 | 1.05 | 2.46 | 7.19 | 18.90 | 10.03 | NA | 0.3 | 13.1 | 0.3 | 18.5 | 0.3 | 8200 | 21.12 | 328.79 | 0.3 | 39.45 |
| 29 | 1.1 | 1.51 | 11.27 | 30.3 | 39.45 | 23.85 | 22.5 | 14.8 | 14.6 | NA | NA | NA | 1200 | 63.26 | 30.11 | 3.32 | 39.45 |
| 30 | 0.62 | 1.52 | 21.45 | 46.6 | 63 | 33.2 | NA | 0.7 | 29.2 | 0.3 | 0.3 | 0.3 | 82.1 | 101.38 | 23.67 | 4.22 | 63 |
| Ctrl-1 | 0.35 | 0.14 | 0.82 | 0 | 0.54 | 43.95 | 31.1 | NA | 0.3 | NA | 0.3 | 0.3 | 84.9 | 1.41 | NA | NA | NA |
| Ctrl-2 | 0.05 | 0.3 | 1.16 | 0.36 | 0.76 | 36.2 | NA | NA | 0.3 | NA | 0.3 | 0.3 | 110 | 2.23 | NA | NA | NA |
| Ctrl-3 | 0.655 | 0.63 | 0.55 | 0.66 | 0.455 | 22.55 | NA | NA | 0.3 | NA | 0.3 | 0.3 | 15.4 | 2.40 | NA | NA | NA |
| Ctrl-4 | 0.565 | 0.35 | 0.305 | 0.42 | 0.63 | 21.15 | NA | NA | 0.3 | NA | 0.3 | 0.3 | 9.6 | 1.67 | NA | NA | NA |

Specific IgE negative subjects were given a value of “0.3”, peanut sIgE >100KU/L was given a value of “101”.

NA: data not available

**Table E6.** Summary of PCB activation tests and sIgE measure for peanut sensitized but tolerant subjects (Group 5). Specific IgE to peanut components were measured using ISAC (ISU-E), Specific IgE negative subjects were given a value of “0.3”.

|  | PCB activation test (%CD63+ cells) | | | | | | sIgE (ISU-E) | | | | | |
| --- | --- | --- | --- | --- | --- | --- | --- | --- | --- | --- | --- | --- |
| ID | ctrl | 1 (ng/ml) | 10 (ng/ml) | 100 (ng/ml) | 1000 (ng/ml) | anti-IgE | Ara h1 | Ara h2 | Ara h3 | Ara h6 | Ara h8 | Ara h9 |
| PT-4 | 0.33 | 0.19 | 0.65 | 1.74 | 1 | 32 | 0.3 | 0.3 | 0.3 | 2.7 | 0.3 | 0.3 |
| PT-2 | 0.41 | 0.36 | 0.43 | 0.37 | 0.33 | 38.4 | 0.48 | 1.27 | 0.3 | 0.76 | 0.3 | 1.8 |
| PT-5 | 0.56 | 0.49 | 0.46 | 0.43 | 0.51 | 40.7 | 0.3 | 0.3 | 1.6 | 0.3 | 0.3 | 0.37 |
| PT-1 | 0.49 | 0.51 | 0.71 | 0.75 | 0.32 | 37.1 | 0.3 | 0.3 | 0.4 | 0.3 | 0.3 | 0.3 |
| PT-3 | 0.38 | 0.15 | 0.32 | 0.35 | 0.36 | 41.3 | 0.3 | 0.3 | 0.4 | 0.3 | 0.3 | 0.3 |

References

1. Grabenhenrich LB, Reich A, Bellach J, Trendelenburg V, Sprikkelman AB, Roberts G, et al. A new framework for the documentation and interpretation of oral food challenges in population-based and clinical research. Allergy. 2017;72(3):453-61.

2. Diamant Z, Gauvreau GM, Cockcroft DW, Boulet LP, Sterk PJ, de Jongh FH, et al. Inhaled allergen bronchoprovocation tests. The Journal of allergy and clinical immunology. 2013;132(5):1045-55 e6.

3. Cockcroft DW, Davis BE. Methacholine PC20: 1-point formula. Ann Allergy Asthma Immunol. 2007;98(5):498-9.
